# Supplementary material for: Epigenetic scores derived in saliva are associated with gestational age at birth
Source: Clin Epigenetics. 2024 Jun 29;16:84. doi: 10.1186/s13148-024-01701-2 (PMC11218140; doi:10.1186/s13148-024-01701-2)
Supplement: Supplementary file 1 — Additional file1 [file 13148_2024_1701_MOESM1_ESM.docx]

**Epigenetic scores derived in saliva are associated with gestational age at birth**

**Supplementary information**

Katie Mckinnon, Eleanor L.S. Conole, Kadi Vaher, Robert F. Hillary, Danni A. Gadd, Justyna Binkowska, Gemma Sullivan, Anna J. Stevenson, Jill Hall, Amy Corrigan, Lee Murphy, Heather C. Whalley, Hilary Richardson, Riccardo E. Marioni, Simon R. Cox, James P. Boardman

**Contents**

Supplementary eMethods

Supplementary Table 1. Spreadsheet of EpiScores with CpG sites and coefficients

Supplementary Table 2. Standardised component loading of the two principal components

Supplementary Table 3. Frequency of perinatal pro-inflammatory co-exposures

Supplementary Figure 1. Relationship between EpiScores and birth gestational age in regression models with the Scottish Index of Multiple Deprivation as socioeconomic status measure

Supplementary Figure 2. Relationship between EpiScores and an interaction between birth gestational age and socioeconomic status in regression models with the Scottish Index of Multiple Deprivation as socioeconomic status measure

Supplementary Figure 3. Relationship between EpiScores and the Scottish Index of Multiple Deprivation as socioeconomic status measure

Supplementary Figure 4. Relationship between EpiScores and birth gestational age in regression models with maternal education as socioeconomic status measure

Supplementary Figure 5. Relationship between EpiScores and an interaction between birth gestational age and socioeconomic status in regression models with maternal education as socioeconomic status measure

Supplementary Figure 6. Relationship between EpiScores and maternal education as socioeconomic status measure

Supplementary Figure 7. Relationship between EpiScores and birth gestational age in regression models with maternal occupation as socioeconomic status measure

Supplementary Figure 8. Relationship between EpiScores and interaction between birth gestational age and socioeconomic status in regression models with maternal occupation as socioeconomic status measure

Supplementary Figure 9. Relationship between EpiScores and maternal occupation as socioeconomic status measure

Supplementary Table 4. Functional roles of proteins of EpiScores associated with birth gestational age

Supplementary Figure 10. Interaction between afamin EpiScore, birth gestational age, and maternal education

Supplementary Figure 11. Interaction between HGFI EpiScore, birth gestational age, and maternal occupation

Supplementary Table 5. Relationship between EpiScores and socioeconomic status measure in unadjusted models and models adjusted for perinatal inflammatory exposures

Supplementary Table 6. Sensitivity analysis comparing EpiScore models with and without adjustment for maternal factors

**eMethods**

Predictor variables:

- Neighbourhood-level SES. Scottish Index of Multiple Deprivation (SIMD, 2016) was derived from the family’s postcode at time of birth[1] (n=331).
  - Continuous measure, from 1 (most deprived) to 6976 (least deprived). SIMD was chosen because it incorporates multiple dimensions (population characteristics, income, employment, health, education, access to services, crime, and housing)[1], neighbourhood deprivation is consistently associated with child development[2], and SIMD is a tractable tool for policy makers[1].
- Family-level SES. Collected from questionnaires at recruitment.
  - Maternal education: highest educational qualification, as ordered factor measure (n=323).
    - None: no qualifications obtained.
    - Basic high school qualification: National 5s, Standard Grades, GCSEs (General Certificates of Secondary Education) or equivalent. This is divided into 1-4 and >5 qualifications.
    - Advanced high school qualification: Highers, A levels (Advanced levels) or equivalent.
    - College qualification: e.g. National Certificate, Higher National Diploma, Higher National Certificate, vocational qualifications.
    - University undergraduate degree.
    - University postgraduate degree.
  - Maternal occupation: current/most recent occupation, as a factor measure (n=328).
    - Unemployed.
    - Homemaker.
    - Still in full time education.
    - Unskilled: cleaners, porters, messengers.
    - Partly skilled: e.g. bus conductor, postman.
    - Manual skilled: e.g. toolmaker, foreman, ambulance man.
    - Non-manual skilled: e.g. typist, police officer, fireman.
    - Professional: e.g. doctors, lawyers, teachers, managers.

Pro-inflammatory covariates:

- Histological chorioamnionitis (HCA), from placental histology.
- Sepsis, from medical records. Positive blood culture and/or physician decision to treat with five days of antibiotics; early-onset is <72 hours after birth and late-onset is after 72 hours.
- Bronchopulmonary dysplasia (BPD), from medical records. Requirement for respiratory support and/or supplemental oxygen after 36 weeks' gestation.
- Necrotising enterocolitis (NEC), from medical records. Medical (7 days nil by mouth) or surgical management.

Maternal factors:

- Maternal smoking, defined as any smoking during pregnancy, from medical records (parent reported at antenatal booking appointment).
- Maternal diabetes, defined as type 1 diabetes mellitus, type 2 diabetes mellitus, or gestational diabetes mellitus, from medical records.
- Maternal obesity, defined as body mass index (BMI) >30, from medical records (maternal measurements at antenatal booking appointment).
- Mode of delivery, from medical records. Categorised as vaginal delivery (non-instrumental cephalic or breech delivery), instrumental delivery (ventouse or forceps), and caesarean (elective or emergency).

EpiScores from Gadd *et al*[3] not included here:

- EpiScores removed due to unavailable cytosine-phosphate-guanine dinucleotide (CpGs): BCAM, Lysozyme C, MMP2.
- EpiScores removed due to duplication: CXCL10, CXCL11, MMP1, Granzyme A, NTRK3.

**Supplementary Table 1.** **Spreadsheet of EpiScores with CpG sites and coefficients**

Excel spreadsheet: Supplementary_Table_1.xlsx

104 included EpiScores with CpG sites and coefficients required to calculate scores for each individual.

**Supplementary Table 2. Standardised component loading of the two principal components**

| **EpiScore** | **PC 1** | **PC 2** |
| --- | --- | --- |
| ADAMTS | 0.042 | 0.081 |
| Adiponectin | 0.055 | -0.095 |
| Afamin | -0.169 | -0.002 |
| Alpha-L-iduronidase | 0.029 | 0.035 |
| Aminoacylase-1 | -0.162 | -0.004 |
| Beta-2-microglobulin | 0.068 | -0.123 |
| BMP1 | -0.156 | 0.104 |
| CCL11 | 0.005 | -0.164 |
| CCL17 | -0.046 | 0.029 |
| CCL18 | 0.035 | 0.074 |
| CCL21 | -0.034 | -0.064 |
| CCL22 | -0.048 | -0.144 |
| CCL25 | -0.112 | 0.037 |
| CD163 | -0.101 | -0.111 |
| CD209 | 0.191 | -0.039 |
| CD48 | -0.099 | -0.081 |
| CD5L | 0.094 | -0.162 |
| CD6 | -0.085 | -0.190 |
| CHIT1 | 0.019 | -0.193 |
| Coagulation factor VII | -0.003 | -0.002 |
| Complement C4 | 0.044 | 0.044 |
| Complement C5a | -0.163 | -0.096 |
| Complement c9 | -0.071 | 0.045 |
| Contactin-4 | 0.088 | 0.114 |
| CRP | -0.138 | 0.013 |
| CRTAM | 0.107 | 0.020 |
| CXCL10 | 0.058 | -0.024 |
| CXCL11 | -0.033 | -0.108 |
| CXCL9 | 0.029 | 0.086 |
| E-selectin | -0.068 | 0.062 |
| Ectodysplasin-A | 0.039 | -0.093 |
| ENPP7 | -0.096 | -0.112 |
| ESM1 | 0.110 | -0.064 |
| Ezrin | 0.108 | -0.188 |
| FAP | 0.092 | 0.008 |
| FCER2 | -0.097 | -0.125 |
| FCGR3A | -0.080 | -0.063 |
| FcRL2 | -0.127 | -0.194 |
| FGF21 | -0.050 | -0.113 |
| Galectin-4 | 0.142 | -0.092 |
| GCSF | 0.106 | -0.005 |
| GDF15 | -0.040 | 0.106 |
| GDF8 | 0.168 | -0.044 |
| GHR | -0.009 | -0.087 |
| GPIba | 0.181 | -0.040 |
| Granulysin | -0.033 | -0.138 |
| Granzyme A | 0.065 | -0.191 |
| HCII | 0.021 | 0.055 |
| HGF | -0.055 | 0.020 |
| HGFA | -0.160 | 0.097 |
| HGFI | -0.002 | -0.006 |
| ICAM5 | 0.132 | -0.087 |
| IGFBP1 | 0.081 | -0.010 |
| IGFBP4 | -0.115 | -0.130 |
| IL19 | -0.064 | -0.073 |
| IL6 | 0.009 | -0.167 |
| Insulin receptor | -0.102 | -0.143 |
| L-selectin | 0.172 | 0.136 |
| Lactotransferrin | 0.022 | 0.031 |
| LGALS3BP | -0.032 | -0.110 |
| LY9 | -0.030 | -0.039 |
| Lymphotoxin | 0.131 | -0.068 |
| MIA | -0.036 | -0.125 |
| MMP1 | -0.176 | 0.007 |
| MMP12 | -0.026 | -0.141 |
| MMP9 | -0.056 | 0.086 |
| MRC2 | -0.107 | -0.086 |
| Myeloperoxidase | -0.112 | 0.092 |
| NCAM1 | 0.151 | -0.108 |
| NCDase | 0.022 | 0.004 |
| Neprilysin | 0.009 | -0.106 |
| NMNAT1 | 0.171 | -0.037 |
| NOTCH1 | 0.128 | 0.030 |
| NTproBNP | -0.057 | 0.136 |
| NTRK3 | 0.022 | 0.152 |
| Oncostatin-M | 0.166 | 0.046 |
| Osteomodulin | -0.027 | 0.037 |
| Pappalysin-1 | 0.114 | 0.060 |
| PIGR | -0.019 | -0.033 |
| RARRES2 | 0.147 | -0.043 |
| Resistin | 0.155 | 0.117 |
| S100A12 | 0.129 | 0.109 |
| S100A9 | 0.109 | 0.179 |
| SCGF-alpha | -0.050 | 0.180 |
| SCGF-beta | -0.088 | 0.159 |
| Semaphorin-3E | 0.081 | -0.076 |
| SERPINA3 | 0.032 | <0.001 |
| SHBG | 0.123 | -0.032 |
| Sialoadhesin | -0.111 | -0.075 |
| SKR3 | -0.013 | 0.002 |
| SLITRK5 | 0.169 | -0.047 |
| SMPD1 | -0.068 | -0.010 |
| Stanniocalcin-1 | -0.092 | 0.010 |
| Testican-2 | 0.013 | -0.134 |
| TGF alpha | 0.070 | 0.132 |
| Thrombopoietin receptor | 0.164 | -0.075 |
| Thrombospondin-2 | -0.007 | -0.102 |
| TNFRSF17 | -0.156 | 0.108 |
| TNFRSF1B | 0.079 | -0.203 |
| Trypsin-2 | -0.020 | 0.021 |
| Tryptase beta-2 | 0.077 | -0.007 |
| VCAM1 | -0.041 | -0.090 |
| VEGFA | -0.118 | -0.040 |
| WFIKKN2 | 0.097 | -0.026 |

Component loadings for the 104 EpiScores for the two principal components.

PC – principal component

**Supplementary Table 3. Frequency of perinatal pro-inflammatory co-exposures**

| **Exposure** | **Preterm (total n=217)** |
| --- | --- |
| Histological chorioamnionitis | 61/158 (38.6%) |
| Sepsis | 56/217 (25.8%) |
| Bronchopulmonary dysplasia | 66/215 (30.7%) |
| Necrotising enterocolitis | 11/217 (5.5%) |

**Supplementary Figure 1. Relationship between EpiScores and birth gestational age in regression models with the Scottish Index of Multiple Deprivation as socioeconomic status measure**


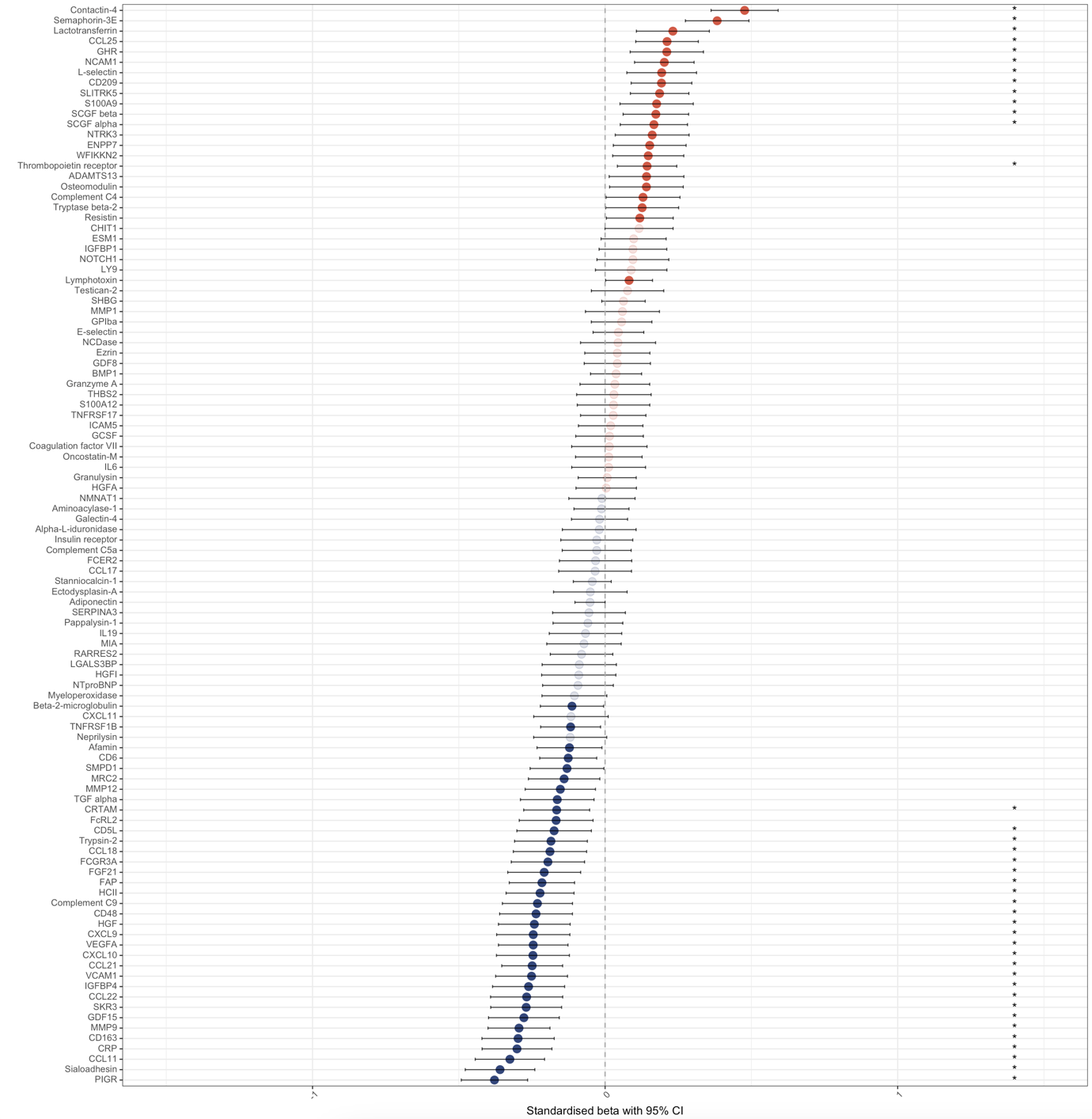


EpiScores associated with birth gestational age in regression models with the Scottish Index of Multiple Deprivation, gestational age at sample, birthweight z-score, sex, and methylation processing batch. n=331. Standardised beta with 95% confidence intervals. Red are positive associations, blue are negative associations. Those with strong colour are individually significant with *p*<0.05 (57/104 EpiScores), and those with an asterisk are significant at the adjusted *p*<8.3x10^-3^ (39/104 EpiScores).

CI – confidence interval

**Supplementary Figure 2. Relationship between EpiScores and an interaction between birth gestational age and socioeconomic status in regression models with the Scottish Index of Multiple Deprivation as socioeconomic status measure**


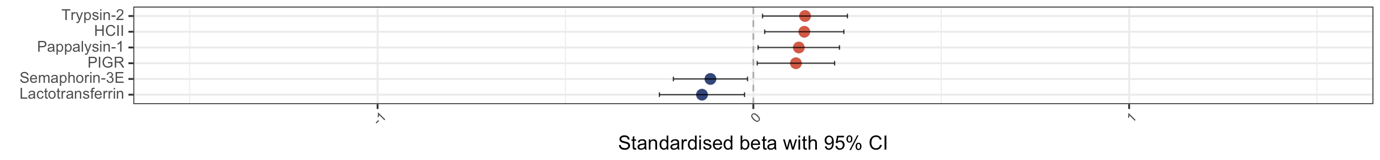


EpiScores associated with an interaction between birth gestational age and the Scottish Index of Multiple Deprivation in regression models with the Scottish Index of Multiple Deprivation, gestational age at birth, gestational age at sample, birthweight z-score, sex, and methylation processing batch. n=331. Standardised beta with 95% confidence intervals. Red are positive associations, blue are negative associations. Those with strong colour are individually significant with *p*<0.05 (6/104 EpiScores), and those with an asterisk are significant at the adjusted *p*<8.3x10^-3^ (0/104 EpiScores).

CI – confidence interval

**Supplementary Figure 3. Relationship between EpiScores and the Scottish Index of Multiple Deprivation as socioeconomic status measure**


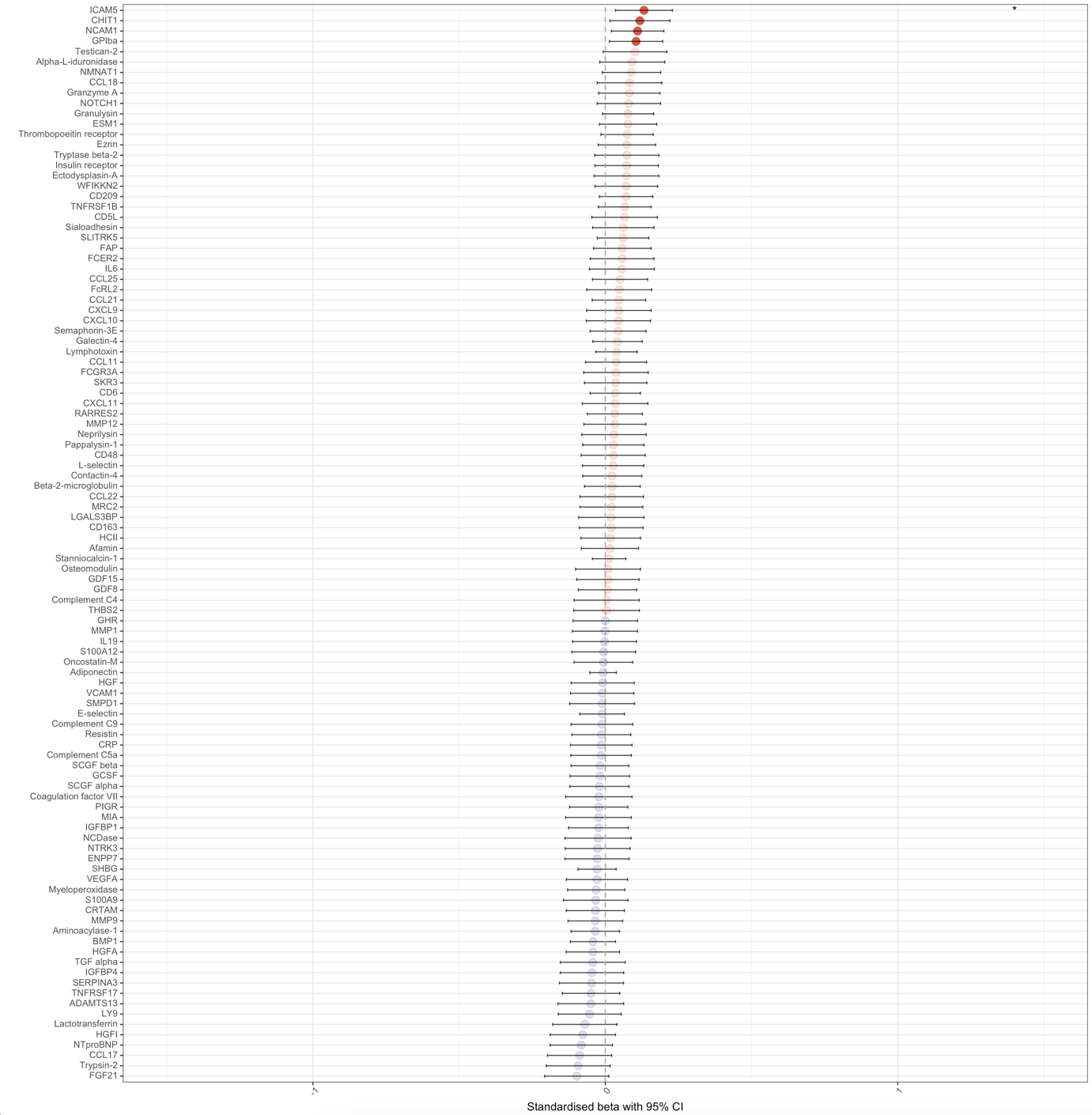


EpiScores associated with the Scottish Index of Multiple Deprivation in regression models with gestational age at birth, gestational age at sample, birthweight z-score, sex, and methylation processing batch. n=331. Standardised beta with 95% confidence intervals. Red are positive associations, blue are negative associations. Those with strong colour are individually significant with *p*<0.05 (4/104 EpiScores), and those with an asterisk are significant at the adjusted *p*<8.3x10^-3^ (1/104 EpiScores).

CI – confidence interval

**Supplementary Figure 4. Relationship between EpiScores and birth gestational age in regression models with maternal education as socioeconomic status measure**


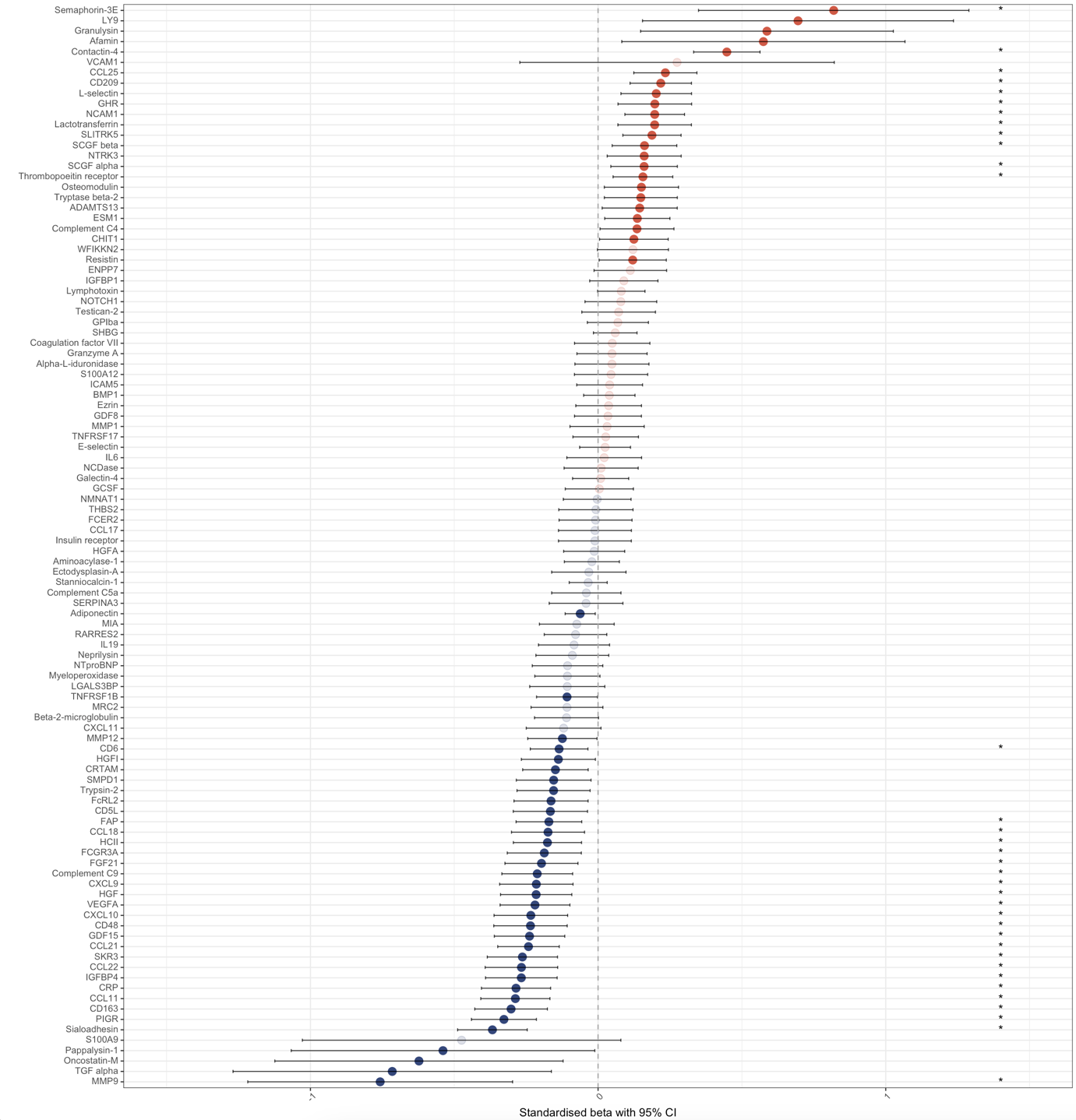


EpiScores associated with birth gestational age in regression models with maternal education, gestational age at sample, birthweight z-score, sex, and methylation processing batch. n=323. Standardised beta with 95% confidence intervals. Red are positive associations, blue are negative associations. Those with strong colour are individually significant with *p*<0.05 (58/104 EpiScores), and those with an asterisk are significant at the adjusted *p*<8.3x10^-3^ (35/104 EpiScores).

CI – confidence interval

**Supplementary Figure 5. Relationship between EpiScores and an interaction between birth gestational age and socioeconomic status in regression models with maternal education as socioeconomic status measure**


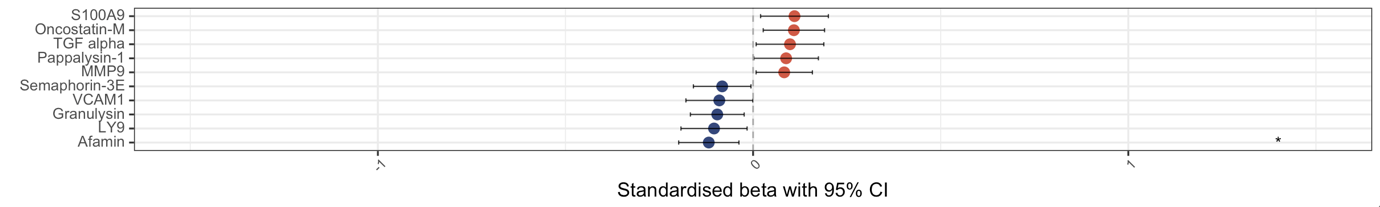


EpiScores associated with an interaction between birth gestational age and maternal education in regression models with maternal education, gestational age at birth, gestational age at sample, birthweight z-score, sex, and methylation processing batch. n=323. Standardised beta with 95% confidence intervals. Red are positive associations, blue are negative associations. Those with strong colour are individually significant with *p*<0.05 (10/104 EpiScores), and those with an asterisk are significant at the adjusted *p*<8.3x10^-3^ (1/104 EpiScores).

CI – confidence interval

**Supplementary Figure 6. Relationship between EpiScores and maternal education as socioeconomic status measure**


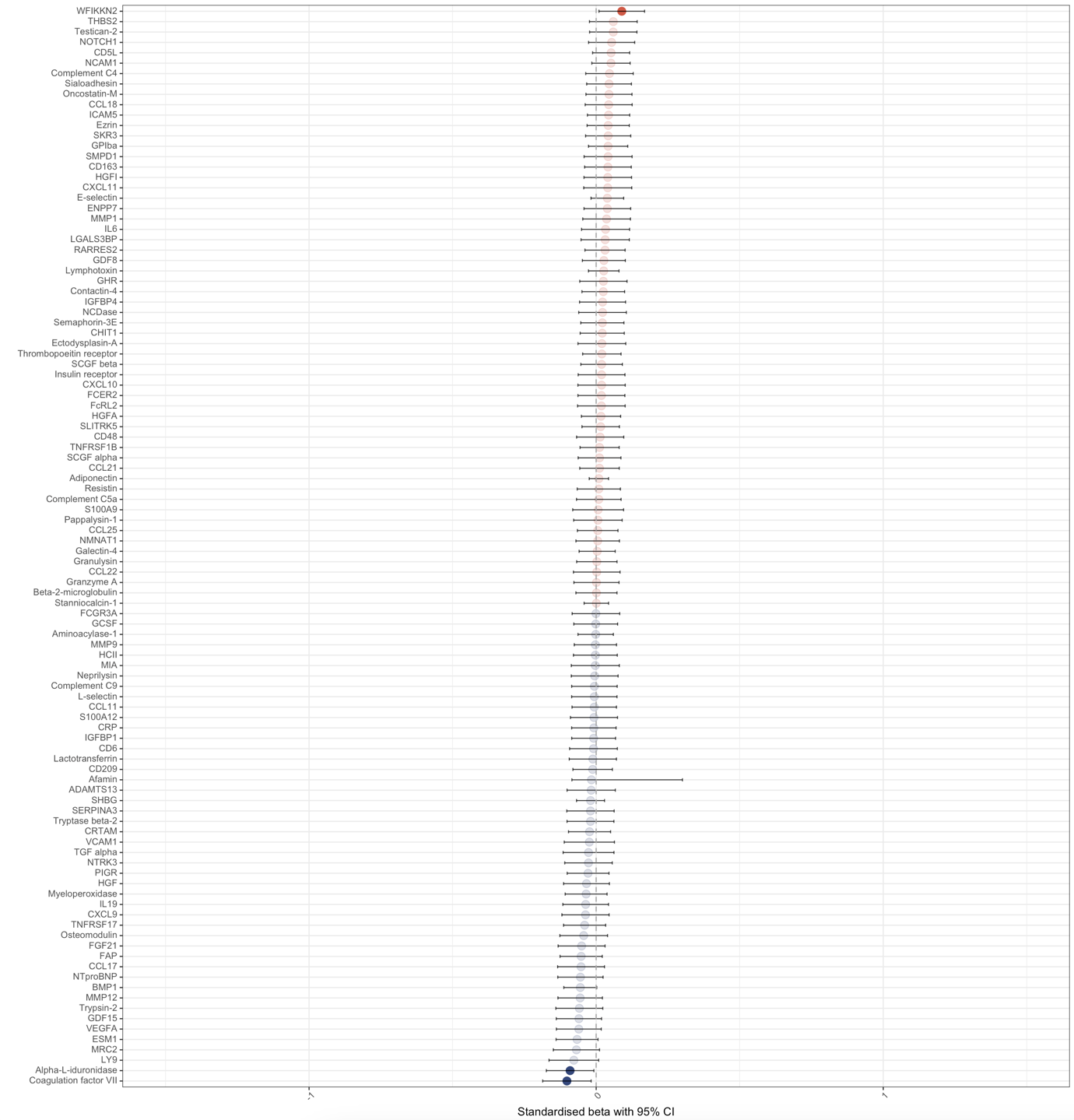


EpiScores associated with maternal education in regression models with the gestational age at birth, gestational age at sample, birthweight z-score, sex, and methylation processing batch. n=323. Standardised beta with 95% confidence intervals. Red are positive associations, blue are negative associations. Those with strong colour are individually significant with *p*<0.05 (3/104 EpiScores), and those with an asterisk are significant at the adjusted *p*<8.3x10^-3^ (0/104 EpiScores).

CI – confidence interval

**Supplementary Figure 7. Relationship between EpiScores and birth gestational age in regression models with maternal occupation as socioeconomic status measure**


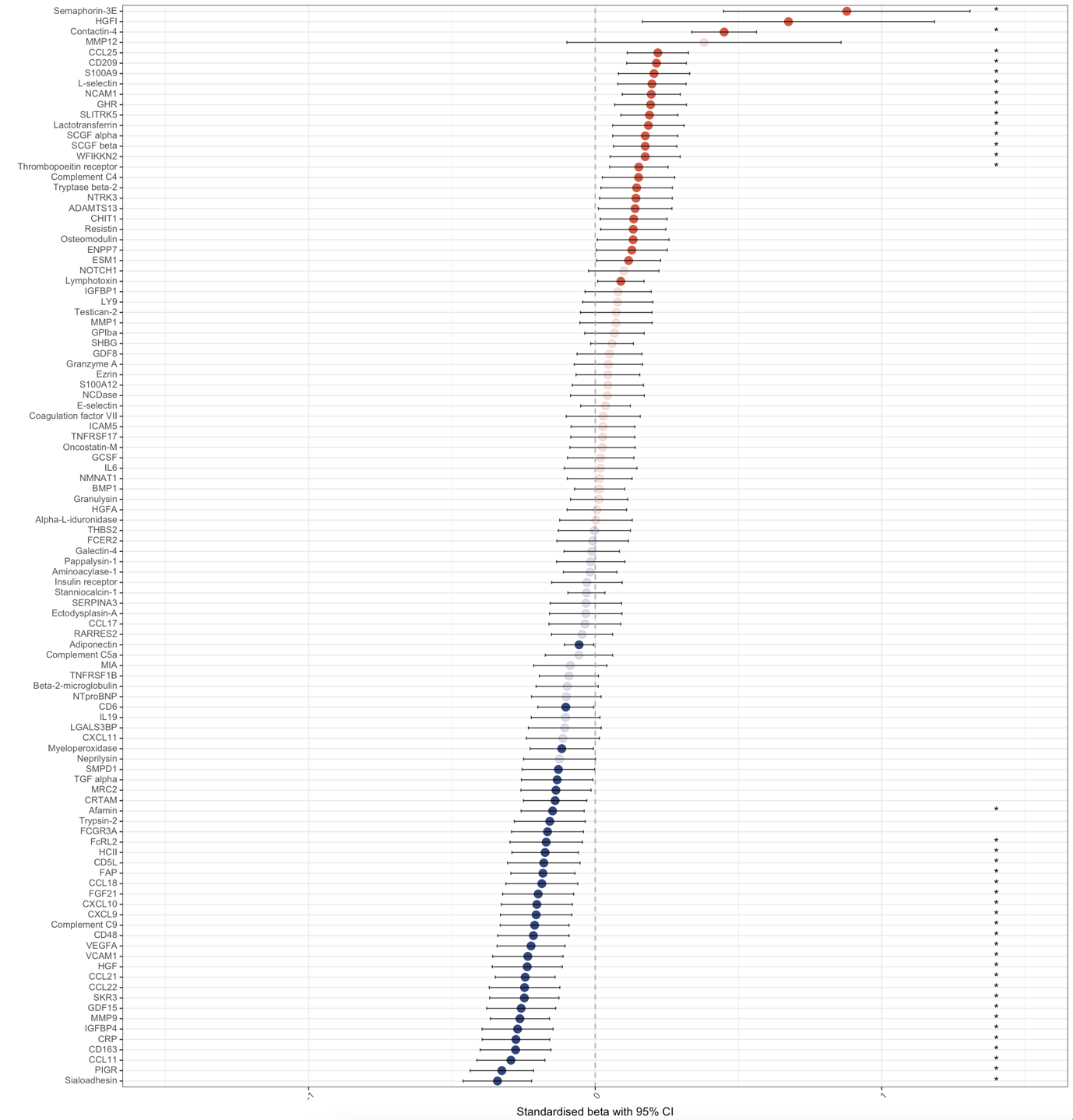


EpiScores associated with birth gestational age in regression models with maternal occupation, gestational age at sample, birthweight z-score, sex, and methylation processing batch. n=328. Standardised beta with 95% confidence intervals. Red are positive associations, blue are negative associations. Those with strong colour are individually significant with *p*<0.05 (59/104 EpiScores), and those with an asterisk are significant at the adjusted *p*<8.3x10^-3^ (39/104 EpiScores).

CI – confidence interval

**Supplementary Figure 8. Relationship between EpiScoresan interaction between birth gestational age and socioeconomic status in regression models with maternal occupation as socioeconomic status measure**


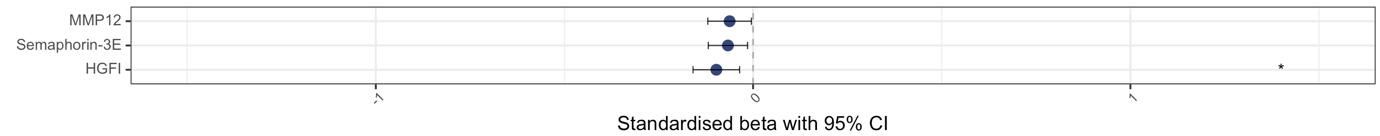


EpiScores associated with an interaction between birth gestational age and maternal occupation in regression models with maternal occupation, gestational age at birth, gestational age at sample, birthweight z-score, sex, and methylation processing batch. n=328. Standardised beta with 95% confidence intervals. Red are positive associations, blue are negative associations. Those with strong colour are individually significant with *p*<0.05 (3/104 EpiScores), and those with an asterisk are significant at the adjusted *p*<8.3x10^-3^ (1/104 EpiScores).

CI – confidence interval

**Supplementary Figure 9. Relationship between EpiScores and maternal occupation as socioeconomic status measure**


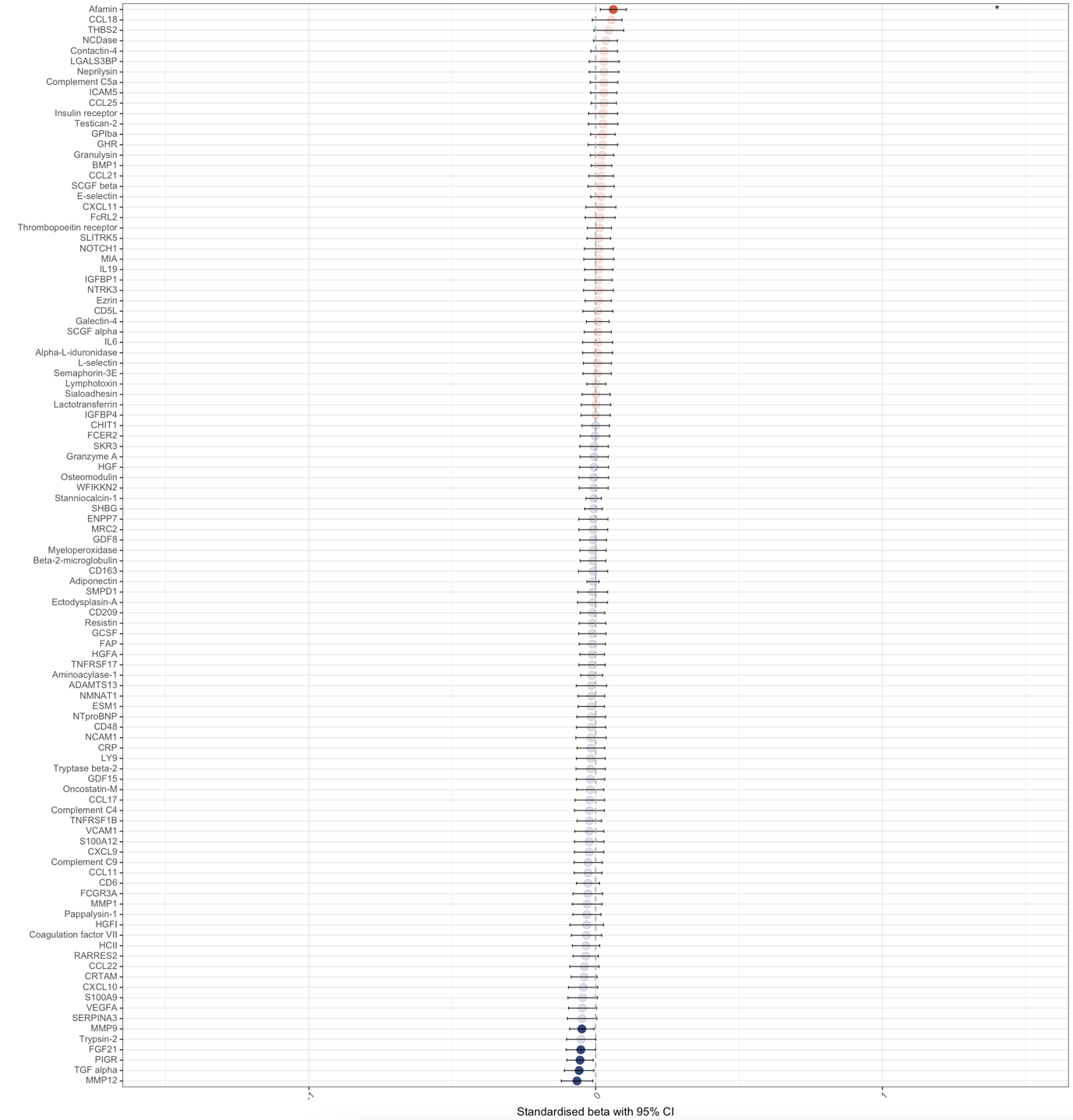


EpiScores associated with maternal occupation in regression models with gestational age at birth, gestational age at sample, birthweight z-score, sex, and methylation processing batch. n=328. Standardised beta with 95% confidence intervals. Red are positive associations, blue are negative associations. Those with strong colour are individually significant with *p*<0.05 (6/104 EpiScores), and those with an asterisk are significant at the adjusted *p*<8.3x10^-3^ (1/104 EpiScores).

CI – confidence interval

**Supplementary Table 4. Functional roles of proteins of EpiScores associated with birth gestational age**

| **EpiScore protein** | **Function and associations** | **Association with birth gestational age** |
| --- | --- | --- |
| Afamin (AFM) | Afamin is a vitamin E binding protein, involved in the transport of vitamin E in body fluids, including across the blood-brain-barrier[4,5], and anti-inflammatory. Afamin EpiScore is associated with diabetes[3], and in pregnant women it afamin is increased with pre-eclampsia and gestational diabetes[6]. In preterm infants, increased afamin is associated with BPD[7]. | Negative |
| CCL11 (C-C motif chemokine 11, eotaxin) | A chemokine involved in the stimulation of eosinophils. CCL11 is involved in allergic responses, but also in psychiatric disorders and Alzheimer’s disease[8–10]. In cord blood, CCL11 concentration is increased with GA[11], and in the presence of vasculopathy and inflammation[12]. CCL11 concentration is increased in blood in infants with BPD[13], patent ductus arteriosus (PDA)[14], and ROP[15], and vitreous fluid in ROP[16]. CCL11 EpiScore is associated with depression and cardiovascular disease in adults[3,17]. | Negative |
| CCL18 (C-C motif chemokine 18) | CCL18 is an anti-inflammatory chemokine, involved in T-cell recruitment and the regulation of other chemokines[18,19]. CCL18 increases after birth in preterm infants[20], and lower CCL18 is associated with intraventricular haemorrhage and cerebral palsy[21,22]. | Negative |
| CCL21 (C-C motif chemokine 21) | CCL21 stimulates immune cell migration and angiogenesis, with a key role in joint inflammation in rheumatoid arthritis[23]. CCL21/CCR7 signalling is involved in the regulation of neuroinflammation[24]. CCL21 is significantly increased in adults and preterm infants with sepsis.[25,26] | Negative |
| CCL22 (C-C motif chemokine 22, MDC, macrophage-derived chemokine) | CCL22 is a macrophage-derived chemokine, expressed in the thymus and chemotactic for monocytes, T-cells, and NK-cells[27]. CCL22 plays a role in neuroinflammation; anti-CCL22 reduces inflammation in an animal model of multiple sclerosis (MS)[28], and CCL22 is raised in adults with first-episode psychosis and correlates with symptoms[29]. CCL22 is involved in allergic conditions[27], and mediates inflammation in haemorrhage-induced lung injury[30]. In an animal model, clarithromycin to prevent preterm birth reduces CCL22 levels.[31] | Negative |
| CCL25 (C-C motif chemokine 25, TECK, thymus-expressed chemokine) | CCL25 is involved in immune function in the thymus, intestine, heart, joints and trachea, and involved in a range of adult inflammatory conditions such as inflammatory bowel disease (IBD), asthma and ischaemic heart disease (IHD)[32]. CCL25 is present in breast milk, particularly colostrum[33]. CCL25 is different between mothers with threatened preterm labour, and those with term infants, although not with those with preterm infants[34]. CCL25 is increased in adults with sepsis, and anti-CCL25 reduces inflammation and lung injury in a mouse model of sepsis[26]. | Positive |
| CD5L (CD5 antigen-like, AIM) | CD5L has multiple anti-inflammatory roles, particularly in the regulation of leukocyte function such as apoptosis inhibition[35]. It is involved in responses to various bacterial infections[35], has been suggested as a treatment for sepsis[36], and is altered by prophylactic antibiotic treatment in an animal model of preterm birth[37]. It has also reduced inflammation in an animal model of ischaemic stroke[38]. | Negative |
| CD6 (T-cell differentiation antigen) | CD6 is a lymphocyte receptor that binds to gram-positive bacteria through lipoteichoic acid, and gram-negative bacteria through lipopolysaccharides (LPS)[39]. CD6 is involved in the early immune response to bacterial sepsis, through immune cell proliferation and function[40], although CD6 is downregulated in sepsis[41]. CD6 also plays a role in auto-immune conditions, such as psoriasis, rheumatoid arthritis, and MS[42]. | Negative |
| CD48 (CD48 antigen) | CD48 is expressed on T-cells and antigen-presenting cells with immune functions[43], and expressed on haematopoeitic stem cells (HSCs) for regulation of stem cells and tumour suppression[44]. It may have roles in allergic airway inflammation, IBD, and bacterial infections[45]. Hypoxia reduced the number of CD48+ HSCs in a mouse model of lung injury[46]. CD48 expression is increased in idiopathic inflammatory myopathies[47]. The presence of CD48 in amniotic fluid is predictive of preterm birth[48], and CD48 gene expression is increased in umbilical cord tissue from preterm infants[49]. | Negative |
| CD163 (Scavenger receptor cysteine-rich type 1 protein M130) | An acute-phase regulated receptor on macrophages, acting as a scavenger-receptor for haemoglobin-haptoglobin complexes, with specific association with a range of inflammatory diseases such as bacterial sepsis, rheumatoid arthritis and IBD[50,51]. CD163 positive cells are higher in placentas from preterm than term infants[52], and in the blood and small intestine mucosa in infants with NEC[53]. In neonates and children with sepsis, those with systemic inflammatory response syndrome have increased expression of CD163[54], and in an animal model, CD163 is involved in PDA closure[55]. CD163 EpiScore was associated with diabetes in adults[3]. | Negative |
| CD209 (DC-SIGN, dendritic cell-specific ICAM-grabbing nonintegrin) | CD209 is expressed on monocyte-derived macrophages, interacting with intercellular adhesion molecules and involved in T-cell mediated immunity[56]. CD209+ cells are present in the decidua during pregnancy[57], and expression in reduced in mothers with spontaneous preterm labour[58]. CD209+ dendritic cells are also increased in inflamed joints in rheumatoid arthritis and psoriatic arthritis, with increased cytokine production[59]. | Positive |
| Complement C9 (CC9) | Complement C9 forms part of the terminal complement complex (TCC) with complement components C5b-9, the membrane attack complex (MAC). TCC is increased in preterm infants in the presence of early onset sepsis[60], and complement C9 is increased in cord blood in the presence of HCA[61]. Deficiencies in C5b-9 complement components increase the risk of certain specific infections such as Neisseria, Haemophilus influenza type B and meningococcus[62]. Complement C9 administration improved survival with Ecoli sepsis in a rat model[63]. However, complement C9 is low in newborn infants compared to adults, increasing after birth from fetal levels[64,65]. Complement C9 expression may be increased in pregnancies leading to spontaneous preterm birth[66], but also rises in labour regardless of prematurity[67]. In term infants with hypoxic ischaemic encephalopathy, cerebrospinal fluid (CSF) complement C9 is reduced and TCC increased, and complement C9 deposition is found across the brain tissues on histopathology[68], and higher TCC levels in CSF correlates with abnormal developmental outcomes[69]. | Negative |
| Contactin-4 (CNTN4, brain-derived immunoglobulin superfamily protein 2, BIG2) | Contactin-4 is an immunoglobulin involved in axon guidance and neurite promotion[70,71]. It is expressed across the central nervous system (CNS), but also in the testis, thyroid, small intestine, and uterus[70,71]. Contactin-4 can also modulate amyloid precursor protein[71]. Contactin-4 increases between birth and term corrected GA in preterm infants[20]. The contactin-4 gene has been suggested as a susceptibility locus for autism[72,73], as well as neuropsychiatric conditions such as anorexia nervosa, Alzheimer’s disease, and schizophrenia[74], and a knock-out mouse model has shown changes in hippocampal synaptic development and changes to memory and stress responses[74]. | Positive |
| CRP (C-reactive protein) | An acute phase inflammatory protein, with actions through activation of the complement pathway, phagocytic cells, and affecting antigen presentation[75]. CRP is increased in preterm infants compared to term infants in cord blood[76] and in blood in the days after birth[77]. Higher concentrations of CRP in the first weeks of life are associated with ventriculomegaly[78], and increased likelihood of executive dysfunction and attention-deficit hyperactivity disorder (ADHD) symptoms at 10 years of age[79,80], although not cerebral palsy[81]. CRP EpiScore is associated with brain structures in adults, in cognitive aging and depression[82,83], as well as with mental health in childhood[84] and psychotic episodes in adulthood[85], and IHD and chronic obstructive pulmonary disease (COPD) in adults[3]. CRP EpiScore is associated with gestational age, perinatal inflammatory processes and neonatal brain development on MRI at term-corrected gestation[86]. | Negative |
| CRTAM (Cytotoxic and regulatory T-cell molecule, Class-I MHC-Restricted T-Cell-Associated Molecule) | CRTAM is an immunoglobulin-superfamily transmembrane protein, expressed on CD4+ and CD8+ T cells, NK cells and NKT cells, playing a role in cellular adhesion, polarity, proliferation and cytokine release[87]. CRTAM is important in intestinal immune responses to parasitic infections particularly[88,89], and its role in other intestinal infections may be related to CRTAM shaping the gut microbiome[90]. | Negative |
| CXCL9 (C-X-C motif chemokine 9, monokine induced by interferon gamma, MIG) | CXCL9 is a chemokine involved in immune cell movement and activation, predominantly regulated by interferon gamma. CXCL9 is involved in the acute and chronic inflammatory response to parasitic infection[91], and raised in fungal infections[25]. In pregnant women with malaria, high CXCL9 levels are associated with pregnancy loss and preterm birth[92]. CXCL9 is low in in women with premature rupture of membranes (PROM)[93], and women with rhesus D alloimmunisation[94], but raised in the mother and placenta in chorioamnionitis[95]. CXCL9 is involved in neuroinflammation, such as MS and the inflammatory response to CNS infections[96]. CXCL9 is also involved in adult inflammatory lung processes such as adenocarcinoma and interstitial lung diseases[97,98]. | Negative |
| CXCL10 (C-X-C motif chemokine 10, interferon gamma-inducible protein 10, IP10) | CXCL10 is chemotactic for monocytes and T-lymphocytes, with a particular role in neuroinflammation, for example MS, Alzheimer’s disease and CNS infections[96,99]. CXCL10 is increased in amniotic fluid in the presence of chronic chorioamnionitis[100], and in mothers with preterm labour[101]. CXCL10 is also involved in the acute and chronic inflammatory response to parasitic infection[91], and interstitial lung disease[98]. In a neonatal animal model, hyperglycaemia stimulates CXCL10[102], and in preterm endothelial cells there is reduced CXCL10 production following pro-inflammatory stimulation compared to term endothelial cells[103]. CXCL10 is low in in women with PROM[93], and women with rhesus D alloimmunisation[94]. In cord blood, increased CXCL10 is associated with an increased likelihood of autism[104]. CXCL10 EpiScore is associated with rheumatoid arthritis in adults[3]. | Negative |
| FAP (Fibroblast activation protein alpha, seprase) | FAP is a type-2 transmembrane serine protein, expressed on fibroblasts, melanocytes, macrophages, and in various malignant cell types[105]. FAP is involved in wound healing, liver and lung fibrosis, osteoarthritis, rheumatoid arthritis, IHD, and a range of malignancies[105]. It inactivates FGF21, so may also be involved in metabolic conditions[105]. FAP is increased in placentas with pre-eclampsia[106], and in the cord blood of preterm neonates after gestational diabetes[107]. FAP increases then decreases after birth in preterm infants[20]. | Negative |
| FCGR3B (Low affinity immunoglobulin gamma Fc region receptor III-B, CD16b) | FCGR3B is a binding site for polymeric immunoglobulin and immune complexes on neutrophils, eosinophils and basophils[108]. FCGR3B is significantly increased in preterm infants with sepsis[25], and in the placenta in chorioamnionitis[95]. The presence of FCGR3B in amniotic fluid is predictive of preterm birth[48]. FCGR3B is involved in systemic sclerosis and systemic lupus erythematosus (SLE)[109], rheumatoid arthritis[110], ulcerative colitis[111], glomerulonephritis[112], and susceptibility to malaria[113]. | Negative |
| FcRL2 (Fc receptor-like protein 2) | FcRL2 is a receptor for the constant region of immunoglobulins, inhibiting B-cell receptor activation and affecting the circulating levels of immune complexes[114,115]. FcRL2 expression is reduced in MS patients with increased neurodegeneration[116]. FcRL2 expression correlates with serum inflammatory markers and disease activity in rheumatoid arthritis[117], and is a prognostic marker in chronic lymphocytic leukaemia[118]. In children with obesity, FcRL2 expression positively correlates with muscle strength[119]. | Negative |
| FGF21 (Fibroblast growth factor 21) | FGF21 is a hormone involved in various metabolic pathways, particularly within skeletal muscle, including acting as a pro-inflammatory cytokine, correlating with other inflammatory proteins such as GDF15 and IL6[120]. FGF21 expression is increased following preterm birth[20], and raised levels are associated with postnatal growth failure[121]. FGF21 is also associated with fetal death or preterm birth in pregnancies with fetal growth restriction[122]. | Negative |
| GDF15 (Growth and differentiation factor 15) | GDF15 is also known as macrophage inhibitory cytokine 1 (MIC-1), part of the transforming growth factor beta (TGF$\beta$)[123,124]. GDF15 is secreted by the placenta, with higher levels with lower gestational age at birth[125], and levels drop with postnatal age in extremely preterm infants, particularly over the first week of life[20,125,126]. GDF15 also correlates with NTproBNP and lactate in this first week of life[126]. It has been associated with adult chronic inflammatory processes in animal models, including atherosclerosis and rheumatoid arthritis.[123] Other predominantly adult disease links include sepsis[127], pre-eclampsia[128], mitochondrial disorders (this study also included children)[129], and Alzheimer’s[130]. In preterm infants, GDF15 has particularly been linked to BPD[131,132]. Higher GDF15 levels are associated with a longer need for mechanical ventilation, prolonged respiratory support need, and length of hospital stay[125]. It has even been suggested that sex differences in GDF15 activation by hyperoxia could explain sex differences in BPD rates, although only in animal models[133]. Other relevant associations are to PDAs in preterm infants[134], and pulmonary hypertension in children[135]. It decreases between birth and term corrected GA in preterm infants[20]. | Negative |
| GHR (Growth hormone receptor) | The primary role of GHR is related to growth and metabolism[136]. GHR is correlated with body mass index (BMI)[137]. Preterm infants with the d3-variant of GHR have increased postnatal catch-up growth[138]. GHR is also present across all body tissues, including throughout the brain, and has a role in neuronal and glial proliferation and differentiation[139]. GH is also protective in animal models of hypoxic ischaemic encephalopathy[139]. GH also mediates pain in a neonatal animal model[140]. GHR knockout mice have increased longevity, and reduced inflammatory cytokines[141], with GH having other roles in inflammatory modulation such as macrophage activation[142]. | Positive |
| HCII (Heparin cofactor 2, HEP2, SERPIND1) | HCII is a serine proteinase inhibitor, inactivating thrombin, particularly in the presence of dermatan sulphate. Levels are low in term infants compared to adults, and even lower in preterm infants[143]. HCII is involved in host defence against gram-negative bacteria and in wound response[144,145]. High HCII levels are protective against in-stent restenosis and atherosclerosis[146]. HCII also plays a role in glucose homeostasis, with HCII levels negatively correlated with HbA1c and insulin resistance[147], and HCII is increased in the cord blood of preterm neonates after gestational diabetes[107]. HCII expression is increased in pregnancies leading to spontaneous preterm birth[66]. | Negative |
| HGF (Hepatocyte growth factor) | HGF mediates inflammation through actions on various cells, and their antigens and cytokines[148]. Activated HGF (although not total HGF) is increased in cord blood for preterm infants compared to term infants, and HGF increases in the early postnatal period in preterm infants[149]. HGF is also elevated in women with post-partum depression[150]. HGF is involved in alveologenesis, and lower HGF levels are associated with more severe BPD in preterm infants[151], with HGF treatment reducing hyperoxia-induced BPD in a mouse model[152]. | Negative |
| IGFBP4 (Insulin-like growth factor-binding protein 4) | IGFBPs form a complex with IGFs in the circulation, with IGFBP4 inhibiting their effects, and present in serum follicular fluid, seminal fluid, interstitial fluid and synovial fluid[153]. One role of IGFBP4 is related to growth and metabolism[136]. Early pregnancy high IGFBP4 is associated with the development of fetal growth restriction[154]. IGFBP4 is increased by inflammatory cytokines in an animal model of lung injury[155], and is involved in immune responses through IGF1 and the balance between T helper 17 and regulatory T cells[156]. It is also a mediator of acute and chronic stress, with reactions to various genotoxic exposures and mediating aging actions[157]. | Negative |
| L-selectin (SELL, CD62L) | L-selectin is a cell adhesion molecule present on leukocytes, with roles in immune cell adhesion, migration, and signal transduction[158]. It may have a particular role in response to virus infection[158], and is raised on day 7 in preterm infants who develop BPD[159]. It is decreased in neonates with severe hypoxia[160]. L-selectin gene expression is increased in umbilical cord tissue from preterm infants[49], but decreased in the cord blood of preterm neonates after gestational diabetes[107]. L-selectin increases between birth and term corrected GA in preterm infants[20]. | Positive |
| Lactotransferrin (Lactoferrin) | Lactotransferrin is an iron-binding glycoprotein, present in tears, saliva, bile, pancreatic fluid, vaginal secretions, semen, and milk[161]. It supports bacterial and viral immune responses, through direct effects and cytokine stimulation[162]. Lactotransferrin supplementation has been clinically investigated, and reduces rates of late-onset sepsis preterm infants, but does not reduce NEC alone on systematic review[163]. Lactotransferrin has roles in neuroinflammation, with involvement in Alzheimer’s disease and Parkinson’s disease[162], and neuroprotection in animal models of neonatal hypoxic ischaemic encephalopathy and preterm brain injury[162], but thus far clinical supplementation does not appear to improve neurodevelopment[163]. Lactotransferrin also regulates bone formation through osteoblasts[164]. | Positive |
| MMP9 (Matrix metalloproteinase-9) | MMP9 is part of a family of enzymes involved in angiogenesis, cell migration, and invasion, and expression is stimulated by various growth factors and cytokines[165]. It plays a role in brain development through the extracellular matrix[166]. MMP9 is decreased in cord blood[76,167] but increased in blood in the days after birth[77], and in CSF in the first weeks of life[168] in preterm infants compared to term infants. MMP9 gene expression is increased in umbilical cord tissue from preterm infants[49]. It is also increased in vitreous and tear samples of babies with ROP[169]. Concentrations of MMP9 in the first weeks of life are also associated with ventriculomegaly[78], and increased likelihood of executive dysfunction and ADHD symptoms at 10 years of age[79,80], although not cerebral palsy[81]. MMP9 is decreased in the CSF of infants with post-haemorrhagic ventricular dilatation (PHVD)[170]. MMP9 is a urinary biomarker of prematurity-associated lung disease in school-age children[171]. MMP9 EpiScore has been associated with rheumatoid arthritis, lung cancer, cardiovascular disease, and COPD in adults[3,17]. | Negative |
| NCAM1 (Neural cell adhesion molecule 1) | NCAM1 is a synaptic adhesion molecule, part of the immunoglobulin superfamily, involved in neural cell differentiation and migration, neurite outgrowth, synaptic plasticity, and signalling[172]. NCAM1 is decreased in the CSF of infants with PHVD[170], and negatively correlated with neurodevelopment[173]. It has been associated with a range of neuroinflammatory processes such as autism[174], bipolar disorder[175], and schizophrenia[176], and is a mediator of stress structural and functional changes in the brain[177]. NCAM1 is differentially expressed in the placenta with spontaneous miscarriage[178], and preterm birth[179]. NCAM1 also correlates with BMI[137], and raised in the urine of preterm babies with non-infectious respiratory disease compared to infections respiratory disease or control[180]. | Positive |
| PIGR (Polymeric immunoglobulin receptor) | PIGR is the receptor for polymeric IgA and IgM in epithelial cells, leading to secretory IgA and IgM release as part of the immune response to infections, increased by inflammatory cytokines[181,182]. Expression of PIGR and an individual’s microbiome are bidirectionally related[183]. PIGR is also upregulated in children with PDA[184]. | Negative |
| S100A9 (Calgranulin B, MRP14) | S100A9 is a calcium binding protein, expressed in neutrophils and monocytes, stimulating leukocyte recruitment and cytokine release during an inflammatory response, and combining with S100A8 to form calprotectin[185]. S100A9 is involved in bacterial and viral infections, metabolic inflammation such as obesity, autoimmune conditions such as psoriatic arthritis and SLE, and Alzheimer’s disease[185]. S100A9 gene expression is increased in umbilical cord tissue from preterm infants[49], and in maternal serum during pregnancies leading to preterm birth[186]. Calprotectin regulates the development of the microbiome, and is lower in preterm infants[187]. | Positive |
| SCGF alpha (Stem cell growth factor alpha, CLEC11A, C-type lectin domain containing 11a, osteolectin) | SCGFs are positive regulators of cell proliferation, both haematopoietic growth factors, and promoters of osteogenesis[188]. CLEC11A is a urinary biomarker of prematurity-associated lung disease in school-age children[171]. Although a specific immune function for CLEC11A is not currently known, other C-type lectin family members have extensive roles in immunity and homeostasis[189]. SCGF alpha is associated with pulmonary vascular disease in preterm infants[190]. SCGF alpha EpiScore is associated with IBD[3]. | Positive |
| SCGF beta (Stem cell growth factor beta, CLEC11A, C-type lectin domain containing 11a, osteolectin) | SCGFs are positive regulators of cell proliferation, both haematopoietic growth factors, and promoters of osteogenesis[188]. CLEC11A is a urinary biomarker of prematurity-associated lung disease in school-age children[171]. Although a specific immune function for CLEC11A is not currently known, other C-type lectin family members have extensive roles in immunity and homeostasis[189]. SCGF beta is increased in idiopathic inflammatory myopathies[47]. SCGF beta is associated with pulmonary vascular disease in preterm infants[190]. | Positive |
| Semaphorin 3E (SEMA3E) | Semaphorin 3E binds to plexin D1 to modulate the immune response through cell migration and proliferation, and cytokine release, particularly in LPS-induced inflammation[191]. Semaphorin 3E also regulates neuron axonal growth, particularly in the development of the hippocampus[192], and is anti-angiogenic so has been proposed as a therapeutic option for ROP[193,194]. It also has roles in malignancies[195] and immune conditions such as systemic sclerosis, asthma[195], and ulcerative colitis[196]. Semaphorin 3E mutations have also been found in CHARGE syndrome[197]. Semaphorin 3E EpiScore is reduced in COPD[3]. | Positive |
| Sialoadhesin (SIGLEC1, CD169) | Sialoadhesin is a sialic acid-binding protein expressed on leukocytes, with a role stimulating immune responses, such as to bacterial and viral infections[198,199], but also autoimmune conditions such as SLE[200], MS[201], and congenital heart block[202]. | Negative |
| SKR3 (Serine/threonine-protein kinase receptor R3, ACVRL1, Activin receptor-like type 1) | SKR3 is a receptor for transforming growth factor beta (TGF-beta) ligands, with its main role in angiogenesis and vascular regulation. SKR3 mutations lead to hereditary haemorrhagic telangiectasia type 2[203], and it is involved in development of the neonatal lung[204], and in Alzheimer’s disease progression[205]. SKR3 EpiScore is associated with depression and COPD[3]. | Negative |
| SLITRK5 (SLIT and NTRK-like protein 5) | SLITRK5 is a transmembrane protein expressed across the CNS, involved in axon and dendrite growth, neuron differentiation and synaptogenesis[206]. It has been linked to autism, ADHD, obsessive compulsive disorder, and Parkinson’s disease[206]. It is also involved in the regulation of osteoblasts[207], and is downregulated in the placentas from infants with neonatal opioid withdrawal syndrome[208]. | Positive |
| Thrombopoietin receptor (TPOR, MPL, myeloproliferative leukaemia protein) | The thrombopoietin receptor regulates platelet production[209], and low expression in neonates and preterm infants specifically leads to thrombocytosis[210]. Thrombopoietin receptor EpiScore is associated with diabetes[3]. | Positive |
| Trypsin-2 (PRSS2, TRY2, protease serine 2) | Trypsin-2 is produced by the pancreas, and upregulated in pancreatitis[211], and IBD[212]. Trypsin-2 is increased in the tracheal aspirates of infants developing BPD[159]. Low trypsin-2 in pregnancy is predictive of developing pre-eclampsia[213]. Trypsin-2 EpiScore is associated with diabetes, COPD and IHD[3]. | Negative |
| VCAM1 (Vascular cell adhesion protein 1) | VCAM1 is involved in leukocyte-endothelial cell adhesion, playing a role in immune responses through leukocyte migration[214]. In adults, it is involved in various inflammatory diagnoses including rheumatoid arthritis, asthma, transplant rejection, and cancer[215]. VCAM1 concentrations are increased in CSF in preterm infants compared to term infants[216], and in blood concentrations vary by birth GA and chronological age but are not associated with placental inflammation[217]. VCAM1 gene expression is increased in umbilical cord tissue from preterm infants[49]. Lower concentrations of VCAM1 in the first weeks of life are associated with increased likelihood of executive dysfunction at 10 years of age[79], and higher concentrations of VCAM1 with echogenic brain lesions[78] and an increased likelihood of ADHD symptoms[80], although not cerebral palsy[81]. In cord blood, increased VCAM1 is associated with an increased likelihood of autism[104]. VCAM1 EpiScore is associated with cardiovascular disease in adults[17]. | Negative |
| VEGFA (Vascular endothelial growth factor A) | VEGF is a proinflammatory protein involved in angiogenesis and vasculogenesis through endothelial cell proliferation and migration, as well as endothelial permeability, and is stimulated by proinflammatory cytokines[218,219]. VEGF concentrations vary by birth GA and chronological age but are not associated with placental inflammation[217]. VEGFA gene expression is increased in umbilical cord tissue from preterm infants[49]. Raised cord blood VEGF is associated with a reduced likelihood of postnatal growth failure[220]. VEGF is increased in blood, vitreous and tear samples of babies with ROP[15,169], and indeed anti-VEGF monoclonal antibodies are used in the treatment of ROP[221]. Lower serum VEGF is seen in preterm infants with respiratory distress syndrome[222], and VEGF has also been linked to BPD, although primarily in animal studies[223]. Concentrations of VEGFR1 and VEGFR2, but not VEGF, in the first weeks of life are associated with ventriculomegaly[78] and increased likelihood of executive dysfunction at 10 years of age[79], although concentrations of VEGF, VEGFR1 and VEGFR2 are all associated with ADHD symptoms[80]. However, concentrations were not associated with cerebral palsy[81]. VEGFA EpiScore is associated with COPD, cardiovascular disease, and diabetes in adults[3,17], and methylation of the VEGFA gene region changed around the time of NEC diagnosis[224]. | Negative |
| WFIKKN2 (WAP, Kazal, immunoglobulin, Kunitz and NTR domain-containing protein 2, GASP1, Growth and differentiation factor-associated serum protein 1) | WFIKKN2 is involved in changing the presentation of transforming growth factor beta family proteins to their receptors[225], including binding to GDF8 and GDF11[226]. There is a bidirectional relationship between WFIKKN2 and obesity in adults[137], and correlates with BMI[137]. WFIKKN2 decreases between birth and term corrected GA in preterm infants[20]. | Positive |

43 EpiScores associated with birth GA in regression models adjusted for socioeconomic status. Roles adapted from the STRING database[227] with additional references as required.

**Supplementary Figure 10. Interaction between afamin EpiScore, birth gestational age, and maternal education**

Relationship between Afamin EpiScore and birth gestational age by maternal education. n=323.

GA – gestational age

**Supplementary Figure 11. Interaction between HGFI EpiScore, birth gestational age, and maternal occupation**

Relationship between HGFI EpiScore and birth gestational age by maternal occupation. n=328.

GA – gestational age, HGFI – hepatocyte growth factor-like protein

**Supplementary Table 5. Relationship between EpiScores and socioeconomic status measure in unadjusted models and models adjusted for perinatal inflammatory exposures**

| **EpiScore** | **Socioeconomic status measure** | **Unadjusted** | | | **Adjusted** | | |
| --- | --- | --- | --- | --- | --- | --- | --- |
|  |  | **Standardised beta (95% CI)** | ***p-*value** | **Adjusted R^2^** | **Standardised beta (95% CI)** | ***p-v*alue** | **Adjusted R^2^** |
| ICAM5 | SIMD | 0.132 (0.035-0.229) | 0.0079 | 0.246 | 0.134 (0.002-0.266) | 0.0454 | 0.325 |
| Afamin | Maternal occupation | 0.061 (0.016-0.107) | 0.0082 | 0.260 | 0.045 (-0.006-0.096) | 0.0827 | 0.391 |
| Afamin | Birth GA* maternal education | -0.11 (-0.198- -0.038) | 0.0041 | 0.262 | 0.014 (-0.082-0.110) | 0.7745 | 0.392 |
| HGFI | Birth GA*  maternal occupation | 0.097 (-0.159- -0.036) | 0.0021 | 0.032 | -0.013 (-0.077-0.051) | 0.6810 | 0.047 |

EpiScores associated with socioeconomic status (Scottish Index of Multiple Deprivation, maternal education, or maternal occupation), or with an interaction between socioeconomic status and birth gestational age. Unadjusted regression models are for all included infants (n=332), and include GA at sample, birthweight z-score, sex, and methylation processing batch. Adjusted regression models are for preterm infants only (n=115), and additionally include inflammatory exposures; sepsis, histological chorioamnionitis, necrotising enterocolitis, and bronchopulmonary dysplasia. Adjusted *p-*value <8.3x10^-3^.

CI – confidence interval, GA – gestational age, HGFI – Hepatocyte growth factor-like protein alpha chain, ICAM5 – Intercellular adhesion molecule 5, SIMD – Scottish Index of Multiple Deprivation.

**Supplementary Table 6. Sensitivity analysis comparing EpiScore models with and without adjustment for maternal factors**

| **EpiScore** | **Predictor** | **Unadjusted** | | | **Adjusted** | | |
| --- | --- | --- | --- | --- | --- | --- | --- |
|  |  | **Standardised beta (95% CI)** | ***p-*value** | **Adjusted R^2^** | **Standardised beta (95% CI)** | ***p-v*alue** | **Adjusted R^2^** |
| *Regression models with SIMD as SES measure* | | | | | | | |
| CCL11 | Birth GA | -0.325 (-0.444 - -0.207) | <0.001 | 0.140 | -0.33 (-0.449 - -0.211) | <0.001 | 0.147 |
| CCL18 | Birth GA | -0.188 (-0.314 - -0.063) | 0.003 | 0.039 | -0.2 (-0.329 - -0.072) | 0.002 | 0.041 |
| CCL21 | Birth GA | -0.249 (-0.353 - -0.145) | <0.001 | 0.335 | -0.247 (-0.353 - -0.141) | <0.001 | 0.333 |
| CCL22 | Birth GA | -0.268 (-0.392 - -0.145) | <0.001 | 0.068 | -0.248 (-0.375 - -0.122) | <0.001 | 0.061 |
| CCL25 | Birth GA | 0.212 (0.105 - 0.319) | <0.001 | 0.295 | 0.214 (0.105 - 0.323) | <0.001 | 0.293 |
| CD163 | Birth GA | -0.297 (-0.421 - -0.174) | <0.001 | 0.064 | -0.314 (-0.44 - -0.187) | <0.001 | 0.074 |
| CD209 | Birth GA | 0.193 (0.089 - 0.296) | <0.001 | 0.340 | 0.204 (0.098 - 0.311) | <0.001 | 0.334 |
| CD48 | Birth GA | -0.236 (-0.361 - -0.112) | <0.001 | 0.048 | -0.236 (-0.364 - -0.108) | <0.001 | 0.044 |
| CD6 | Birth GA | -0.174 (-0.302 - -0.047) | 0.007 | 0.007 | -0.18 (-0.311 - -0.048) | 0.007 | 0.001 |
| Complement C9 | Birth GA | -0.231 (-0.351 - -0.112) | <0.001 | 0.117 | -0.229 (-0.353 - -0.106) | <0.001 | 0.110 |
| Contactin-4 | Birth GA | 0.477 (0.362 - 0.592) | <0.001 | 0.194 | 0.462 (0.343 - 0.58) | <0.001 | 0.184 |
| CRP | Birth GA | -0.301 (-0.421 - -0.182) | <0.001 | 0.126 | -0.286 (-0.408 - -0.164) | <0.001 | 0.112 |
| CRTAM | Birth GA | -0.166 (-0.278 - -0.053) | 0.004 | 0.219 | -0.153 (-0.269 - -0.037) | 0.010 | 0.212 |
| CXCL10 | Birth GA | -0.247 (-0.371 - -0.122) | <0.001 | 0.046 | -0.253 (-0.379 - -0.127) | <0.001 | 0.057 |
| CXCL9 | Birth GA | -0.246 (-0.371 - -0.12) | <0.001 | 0.039 | -0.234 (-0.362 - -0.106) | <0.001 | 0.042 |
| FAP | Birth GA | -0.216 (-0.328 - -0.104) | <0.001 | 0.236 | -0.216 (-0.331 - -0.102) | <0.001 | 0.232 |
| FCGR3B | Birth GA | -0.196 (-0.321 - -0.07) | 0.002 | 0.031 | -0.205 (-0.333 - -0.077) | 0.002 | 0.038 |
| FGF21 | Birth GA | -0.208 (-0.333 - -0.083) | 0.001 | 0.048 | -0.2 (-0.328 - -0.072) | 0.002 | 0.042 |
| GDF15 | Birth GA | -0.278 (-0.399 - -0.157) | <0.001 | 0.102 | -0.257 (-0.382 - -0.133) | <0.001 | 0.097 |
| GHR | Birth GA | 0.211 (0.086 - 0.336) | 0.001 | 0.036 | 0.195 (0.066 - 0.324) | 0.003 | 0.029 |
| HCII | Birth GA | -0.222 (-0.338 - -0.106) | <0.001 | 0.186 | -0.225 (-0.343 - -0.108) | <0.001 | 0.198 |
| HGF | Birth GA | -0.242 (-0.364 - -0.119) | <0.001 | 0.078 | -0.249 (-0.374 - -0.124) | <0.001 | 0.082 |
| IGFBP4 | Birth GA | -0.262 (-0.385 - -0.138) | <0.001 | 0.070 | -0.263 (-0.39 - -0.135) | <0.001 | 0.057 |
| L-selectin | Birth GA | 0.193 (0.074 - 0.313) | 0.002 | 0.131 | 0.182 (0.059 - 0.305) | 0.004 | 0.120 |
| Lactotransferrin | Birth GA | 0.232 (0.107 - 0.356) | <0.001 | 0.060 | 0.234 (0.104 - 0.363) | <0.001 | 0.051 |
| MMP9 | Birth GA | -0.294 (-0.4 - -0.188) | <0.001 | 0.313 | -0.289 (-0.396 - -0.182) | <0.001 | 0.331 |
| NCAM1 | Birth GA | 0.202 (0.101 - 0.304) | <0.001 | 0.364 | 0.194 (0.089 - 0.298) | <0.001 | 0.356 |
| PIGR | Birth GA | -0.378 (-0.492 - -0.265) | <0.001 | 0.223 | -0.379 (-0.494 - -0.264) | <0.001 | 0.240 |
| S100A9 | Birth GA | 0.176 (0.051 - 0.301) | 0.006 | 0.035 | 0.18 (0.051 - 0.31) | 0.007 | 0.027 |
| SCGF alpha | Birth GA | 0.167 (0.052 - 0.282) | 0.005 | 0.186 | 0.168 (0.05 - 0.286) | 0.005 | 0.180 |
| SCGF beta | Birth GA | 0.174 (0.062 - 0.285) | 0.002 | 0.227 | 0.168 (0.053 - 0.283) | 0.004 | 0.219 |
| Semaphorin-3E | Birth GA | 0.383 (0.274 - 0.492) | <0.001 | 0.286 | 0.391 (0.279 - 0.503) | <0.001 | 0.288 |
| Sialoadhesin | Birth GA | -0.359 (-0.478 - -0.24) | <0.001 | 0.131 | -0.355 (-0.477 - -0.232) | <0.001 | 0.118 |
| SKR3 | Birth GA | -0.27 (-0.391 - -0.149) | <0.001 | 0.100 | -0.284 (-0.407 - -0.161) | <0.001 | 0.106 |
| SLITRK5 | Birth GA | 0.186 (0.086 - 0.286) | <0.001 | 0.388 | 0.193 (0.09 - 0.296) | <0.001 | 0.379 |
| Thrombopoietin receptor | Birth GA | 0.143 (0.042 - 0.245) | 0.006 | 0.368 | 0.133 (0.029 - 0.236) | 0.012 | 0.360 |
| Trypsin-2 | Birth GA | -0.185 (-0.309 - -0.061) | 0.004 | 0.060 | -0.163 (-0.29 - -0.035) | 0.013 | 0.048 |
| VCAM1 | Birth GA | -0.251 (-0.374 - -0.128) | <0.001 | 0.071 | -0.261 (-0.386 - -0.135) | <0.001 | 0.066 |
| VEGFA | Birth GA | -0.246 (-0.365 - -0.127) | <0.001 | 0.135 | -0.243 (-0.366 - -0.12) | <0.001 | 0.122 |
| ICAM5 | SIMD | 0.132 (0.035 - 0.23) | 0.008 | 0.246 | 0.12 (0.014 - 0.226) | 0.027 | 0.243 |
| *Regression models with maternal education as SES measure* | | | | | | | |
| CCL11 | Birth GA | -0.288 (-0.408 - -0.167) | <0.001 | 0.129 | -0.303 (-0.424 - -0.182) | <0.001 | 0.141 |
| CCL18 | Birth GA | -0.174 (-0.301 - -0.047) | 0.007 | 0.038 | -0.186 (-0.316 - -0.056) | 0.005 | 0.046 |
| CCL21 | Birth GA | -0.242 (-0.348 - -0.135) | <0.001 | 0.332 | -0.233 (-0.342 - -0.124) | <0.001 | 0.332 |
| CCL22 | Birth GA | -0.266 (-0.392 - -0.14) | <0.001 | 0.070 | -0.244 (-0.373 - -0.115) | <0.001 | 0.065 |
| CCL25 | Birth GA | 0.234 (0.124 - 0.344) | <0.001 | 0.300 | 0.229 (0.117 - 0.341) | <0.001 | 0.296 |
| CD163 | Birth GA | -0.302 (-0.429 - -0.176) | <0.001 | 0.062 | -0.315 (-0.444 - -0.186) | <0.001 | 0.072 |
| CD209 | Birth GA | 0.218 (0.111 - 0.325) | <0.001 | 0.326 | 0.223 (0.113 - 0.332) | <0.001 | 0.319 |
| CD48 | Birth GA | -0.235 (-0.363 - -0.107) | <0.001 | 0.047 | -0.228 (-0.358 - -0.098) | <0.001 | 0.045 |
| CD5L | Birth GA | -0.135 (-0.236 - -0.035) | 0.008 | 0.411 | -0.133 (-0.236 - -0.031) | 0.011 | 0.406 |
| Complement C9 | Birth GA | -0.211 (-0.335 - -0.088) | <0.001 | 0.151 | -0.217 (-0.344 - -0.09) | <0.001 | 0.100 |
| Contactin-4 | Birth GA | 0.448 (0.332 - 0.563) | <0.001 | 0.183 | 0.436 (0.317 - 0.555) | <0.001 | 0.171 |
| CRP | Birth GA | -0.285 (-0.405 - -0.165) | <0.001 | 0.124 | -0.282 (-0.405 - -0.159) | <0.001 | 0.108 |
| CXCL10 | Birth GA | -0.234 (-0.362 - -0.106) | <0.001 | 0.042 | -0.244 (-0.373 - -0.115) | <0.001 | 0.052 |
| CXCL9 | Birth GA | -0.215 (-0.342 - -0.087) | 0.001 | 0.039 | -0.208 (-0.339 - -0.078) | 0.002 | 0.045 |
| FAP | Birth GA | -0.171 (-0.285 - -0.057) | 0.003 | 0.232 | -0.18 (-0.297 - -0.063) | 0.003 | 0.230 |
| FCGR3B | Birth GA | -0.187 (-0.316 - -0.058) | 0.005 | 0.028 | -0.189 (-0.319 - -0.058) | 0.005 | 0.040 |
| FGF21 | Birth GA | -0.197 (-0.324 - -0.07) | 0.002 | 0.041 | -0.186 (-0.316 - -0.057) | 0.005 | 0.036 |
| GDF15 | Birth GA | -0.238 (-0.361 - -0.116) | <0.001 | 0.104 | -0.235 (-0.361 - -0.109) | <0.001 | 0.090 |
| GHR | Birth GA | 0.197 (0.069 - 0.325) | 0.003 | 0.038 | 0.19 (0.059 - 0.321) | 0.005 | 0.035 |
| HCII | Birth GA | -0.176 (-0.295 - -0.057) | 0.004 | 0.156 | -0.187 (-0.307 - -0.067) | 0.002 | 0.170 |
| HGF | Birth GA | -0.215 (-0.339 - -0.091) | <0.001 | 0.068 | -0.223 (-0.348 - -0.097) | <0.001 | 0.082 |
| IGFBP4 | Birth GA | -0.267 (-0.391 - -0.142) | <0.001 | 0.065 | -0.266 (-0.394 - -0.137) | <0.001 | 0.053 |
| L-selectin | Birth GA | 0.202 (0.08 - 0.325) | 0.001 | 0.127 | 0.19 (0.063 - 0.317) | 0.003 | 0.117 |
| Lactotransferrin | Birth GA | 0.197 (0.069 - 0.324) | 0.003 | 0.042 | 0.204 (0.072 - 0.335) | 0.003 | 0.039 |
| MMP9 | Birth GA | -0.758 (-1.218 - -0.297) | 0.001 | 0.303 | -0.73 (-1.198 - -0.263) | 0.002 | 0.317 |
| NCAM1 | Birth GA | 0.197 (0.093 - 0.301) | <0.001 | 0.349 | 0.191 (0.085 - 0.297) | <0.001 | 0.341 |
| PIGR | Birth GA | -0.327 (-0.44 - -0.214) | <0.001 | 0.198 | -0.334 (-0.449 - -0.22) | <0.001 | 0.214 |
| SCGF alpha | Birth GA | 0.16 (0.044 - 0.276) | 0.007 | 0.174 | 0.156 (0.037 - 0.274) | 0.010 | 0.164 |
| SCGF beta | Birth GA | 0.161 (0.049 - 0.274) | 0.005 | 0.218 | 0.151 (0.036 - 0.266) | 0.010 | 0.207 |
| Semaphorin-3E | Birth GA | 0.819 (0.349 - 1.289) | <0.001 | 0.265 | 0.823 (0.338 - 1.309) | <0.001 | 0.264 |
| Sialoadhesin | Birth GA | -0.367 (-0.488 - -0.246) | <0.001 | 0.135 | -0.36 (-0.485 - -0.236) | <0.001 | 0.120 |
| SKR3 | Birth GA | -0.263 (-0.385 - -0.141) | <0.001 | 0.089 | -0.276 (-0.4 - -0.153) | <0.001 | 0.103 |
| SLITRK5 | Birth GA | 0.187 (0.086 - 0.289) | <0.001 | 0.369 | 0.196 (0.0913 - 0.3) | <0.001 | 0.359 |
| Thrombopoietin receptor | Birth GA | 0.156 (0.052 - 0.26) | 0.003 | 0.364 | 0.147 (0.042 - 0.253) | 0.006 | 0.358 |
| VEGFA | Birth GA | -0.219 (-0.341 - -0.098) | <0.001 | 0.132 | -0.224 (-0.35 - -0.099) | <0.001 | 0.120 |
| Afamin | Maternal education x birth GA | -0.118 (-0.198 - -0.038) | 0.004 | 0.262 | -0.108 (-0.189 - -0.026) | 0.010 | 0.265 |
| *Regression models with maternal occupation as SES measure* | | | | | | | |
| Afamin | Birth GA | -0.149 (-0.259 - -0.039) | 0.008 | 0.260 | -0.148 (-0.26 - -0.036) | 0.010 | 0.261 |
| CCL11 | Birth GA | -0.294 (-0.413 - -0.175) | <0.001 | 0.138 | -0.315 (-0.434 - -0.195) | <0.001 | 0.144 |
| CCL18 | Birth GA | -0.186 (-0.312 - -0.06) | 0.004 | 0.038 | -0.197 (-0.326 - -0.067) | 0.003 | 0.039 |
| CCL21 | Birth GA | -0.244 (-0.349 - -0.14) | <0.001 | 0.325 | -0.239 (-0.346 - -0.133) | <0.001 | 0.325 |
| CCL22 | Birth GA | -0.247 (-0.37 - -0.123) | <0.001 | 0.077 | -0.236 (-0.362 - -0.109) | <0.001 | 0.065 |
| CCL25 | Birth GA | 0.219 (0.111 - 0.326) | <0.001 | 0.301 | 0.222 (0.112 - 0.331) | <0.001 | 0.294 |
| CD163 | Birth GA | -0.278 (-0.401 - -0.154) | <0.001 | 0.058 | -0.295 (-0.421 - -0.169) | <0.001 | 0.073 |
| CD209 | Birth GA | 0.214 (0.11 - 0.318) | <0.001 | 0.331 | 0.217 (0.109 - 0.324) | <0.001 | 0.325 |
| CD48 | Birth GA | -0.216 (-0.34 - -0.091) | <0.001 | 0.045 | 0.208 (-0.342 - -0.087) | <0.001 | 0.046 |
| CD6 | Birth GA | -0.18 (-0.306 - -0.053) | 0.006 | 0.008 | -0.186 (-0.317 - -0.055) | 0.006 | 0.001 |
| Complement C9 | Birth GA | -0.211 (-0.331 - -0.091) | <0.001 | 0.119 | -0.217 (-0.341 - -0.093) | <0.001 | 0.112 |
| Contactin-4 | Birth GA | 0.45 (0.337 - 0.563) | <0.001 | 0.195 | 0.44 (0.324 - 0.557) | <0.001 | 0.185 |
| CRP | Birth GA | -0.277 (-0.395 - -0.159) | <0.001 | 0.123 | -0.268 (-0.388 - -0.147) | <0.001 | 0.107 |
| CXCL10 | Birth GA | -0.204 (-0.328 - -0.08) | 0.001 | 0.051 | -0.222 (-0.347 - -0.097) | <0.001 | 0.067 |
| CXCL9 | Birth GA | -0.206 (-0.331 - -0.081) | 0.001 | 0.036 | -0.202 (-0.33 - -0.074) | 0.002 | 0.040 |
| FAP | Birth GA | -0.183 (-0.294 - -0.071) | 0.001 | 0.229 | -0.191 (-0.307 - -0.076) | 0.001 | 0.225 |
| FcRL2 | Birth GA | -0.171 (-0.298 - -0.045) | 0.008 | 0.025 | -0.178 (-0.31 - -0.047) | 0.008 | 0.015 |
| FGF21 | Birth GA | -0.199 (-0.323 - -0.075) | 0.002 | 0.051 | -0.193 (-0.32 - -0.066) | 0.003 | 0.050 |
| GDF15 | Birth GA | -0.258 (-0.379 - -0.138) | <0.001 | 0.098 | -0.246 (-0.37 - -0.122) | <0.001 | 0.088 |
| GHR | Birth GA | 0.193 (0.068 - 0.318) | 0.003 | 0.035 | 0.187 (0.058 - 0.316) | 0.005 | 0.029 |
| HCII | Birth GA | -0.175 (-0.291 - -0.059) | 0.003 | 0.177 | -0.19 (-0.308 - -0.073) | 0.002 | 0.189 |
| HGF | Birth GA | -0.237 (-0.36 - -0.115) | <0.001 | 0.070 | -0.245 (-0.369 - -0.12) | <0.001 | 0.081 |
| IGFBP4 | Birth GA | -0.271 (-0.395 - -0.147) | <0.001 | 0.067 | -0.267 (-0.396 - -0.139) | <0.001 | 0.054 |
| L-selectin | Birth GA | 0.198 (0.079 - 0.317) | 0.001 | 0.127 | 0.187 (0.0641 - 0.311) | 0.003 | 0.115 |
| Lactotransferrin | Birth GA | 0.186 (0.061 - 0.31) | 0.004 | 0.041 | 0.192 (0.063 - 0.321) | 0.004 | 0.033 |
| MMP9 | Birth GA | -0.263 (-0.366 - -0.159) | <0.001 | 0.322 | -0.27 (-0.375 - -0.165) | <0.001 | 0.338 |
| NCAM1 | Birth GA | 0.196 (0.094 - 0.297) | <0.001 | 0.360 | 0.184 (0.08 - 0.288) | <0.001 | 0.354 |
| PIGR | Birth GA | -0.326 (-0.436 - -0.215) | <0.001 | 0.225 | -0.339 (-0.451 - -0.226) | <0.001 | 0.238 |
| S100A9 | Birth GA | 0.205 (0.08 - 0.33) | 0.001 | 0.045 | 0.201 (0.072 - 0.33) | 0.002 | 0.034 |
| SCGF alpha | Birth GA | 0.175 (0.061 - 0.289) | 0.003 | 0.183 | 0.173 (0.056 - 0.29) | 0.004 | 0.174 |
| SCGF beta | Birth GA | 0.175 (0.064 - 0.285) | 0.002 | 0.224 | 0.17 (0.056 - 0.283) | 0.003 | 0.213 |
| Semaphorin-3E | Birth GA | 0.878 (0.448 - 1.308) | <0.001 | 0.287 | 0.909 (0.465 - 1.352) | <0.001 | 0.288 |
| Sialoadhesin | Birth GA | -0.341 (-0.461 - -0.222) | <0.001 | 0.124 | -0.336 (-0.459 - -0.212) | <0.001 | 0.109 |
| SKR3 | Birth GA | -0.247 (-0.368 - -0.126) | <0.001 | 0.098 | -0.269 (-0.392 - -0.146) | <0.001 | 0.102 |
| SLITRK5 | Birth GA | 0.189 (0.09 - 0.289) | <0.001 | 0.376 | 0.197 (0.095 - 0.3) | <0.001 | 0.366 |
| Thrombopoietin receptor | Birth GA | 0.152 (0.05 - 0.254) | 0.004 | 0.363 | 0.141 (0.037 - 0.245) | 0.008 | 0.356 |
| VCAM1 | Birth GA | -0.235 (-0.358 - -0.112) | <0.001 | 0.069 | -0.241 (-0.366 - -0.116) | <0.001 | 0.071 |
| VEGFA | Birth GA | -0.224 (-0.342 - -0.105) | <0.001 | 0.140 | -0.231 (-0.353 - -0.108) | <0.001 | 0.131 |
| WFIKKN2 | Birth GA | 0.174 (0.052 - 0.297) | 0.005 | 0.084 | 0.176 (0.052 - 0.301) | 0.006 | 0.094 |
| Afamin | Maternal occupation | 0.061 (0.016 - 0.107) | 0.008 | 0.260 | 0.0547 (0.003 - 0.107) | 0.039 | 0.261 |
| HGFI | Maternal occupation x birth GA | -0.097 (-0.159 - -0.036) | 0.002 | 0.032 | -0.103 (-0.166 - -0.04) | 0.001 | 0.036 |

EpiScores associated with birth gestational age, socioeconomic status (Scottish Index of Multiple Deprivation, maternal education, or maternal occupation), or with an interaction between socioeconomic status and birth gestational age. Unadjusted regression models include GA at sample, birthweight z-score, sex, and methylation processing batch. Adjusted regression models additionally include maternal factors; maternal smoking, diabetes, obesity, and mode of delivery. Adjusted *p-*value <8.3x10^-3^.

CCL11 – C-C chemokine 11, CCL18 – C-C chemokine 18, CCL21 – C-C chemokine 21, CCL22 – C-C chemokine 22, CCL25 – C-C chemokine 25, CD5L – CD5 antigen-like protein, CD6 – T-cell differentiation antigen, CD163 – scavenger receptor cysteine-rich type 1 protein M130, CI – confidence interval, CRP – C-reactive protein, CRTAM – Cytotoxic and regulatory T-cell molecule, CXCL9 – C-X-C motif chemokine 9, CXCL10 – C-X-C motif chemokine 10, FAP – Fibroblast Activation Protein alpha, FCGR3B – Low affinity immunoglobulin gamma Fc region receptor III-B, FcRL2 – Fc receptor-like protein 2, FGF21 – Fibroblast growth factor 21, GA – gestational age, GDF15 – Growth/differentiation factor 15, GHR – Growth hormone receptor, HCII – Heparin cofactor II, HGF – Hepatocyte growth factor alpha chain, HGFI – Hepatocyte growth factor-like protein alpha chain, ICAM5 – Intercellular adhesion molecule 5, IGFBP4 – Insulin-like growth factor-binding protein 4, MMP9 – Matrix metalloproteinase-9, NCAM1 – Neural cell adhesion molecule 1, PIGR – Polymeric immunoglobulin receptor, SCGF – stem cell growth factor, SIMD – Scottish Index of Multiple Deprivation, SKR3 – Serine/threonine-protein kinase receptor R3, SLITRK5 – SLIT and NTRK-like protein 5, VCAM1 – Vascular cell adhesion protein 1, VEGFA – Vascular endothelial growth factor A, WFIKKN2 – WAP Kazal immunoglobulin Kunitz and NTR domain-containing protein 2.

**References**

1. Scottish National Statistics. SIMD - Scottish Index of Multiple Deprivation: SIMD16 technical notes. Edinburgh: Scottish National Statistics; 2016 p. 1–69.

2. Minh A, Muhajarine N, Janus M, Brownell M, Guhn M. A review of neighborhood effects and early child development: How, where, and for whom, do neighborhoods matter? Health Place. 2017;46:155–74.

3. Gadd DA, Hillary RF, McCartney DL, Zaghlool SB, Stevenson AJ, Cheng Y, et al. Epigenetic scores for the circulating proteome as tools for disease prediction. Elife. 2022;11:e71802.

4. Voegele AF, Jerković L, Wellenzohn B, Eller P, Kronenberg F, Liedl KR, et al. Characterization of the Vitamin E-Binding Properties of Human Plasma Afamin. Biochemistry. 2002;41:14532–8.

5. Kratzer I, Bernhart E, Wintersperger A, Hammer A, Waltl S, Malle E, et al. Afamin is synthesized by cerebrovascular endothelial cells and mediates α‐tocopherol transport across an in vitro model of the blood–brain barrier. J Neurochem. 2009;108:707–18.

6. Tramontana A, Pablik E, Stangl G, Hartmann B, Dieplinger H, Hafner E. Combination of first trimester serum afamin levels and three-dimensional placental bed vascularization as a possible screening method to detect women at-risk for adverse pregnancy complications like pre-eclampsia and gestational diabetes mellitus in low-risk pregnancies. Placenta. 2018;62:9–15.

7. Zasada M, Suski M, Bokiniec R, Szwarc-Duma M, Borszewska-Kornacka MK, Madej J, et al. Comparative two time-point proteome analysis of the plasma from preterm infants with and without bronchopulmonary dysplasia. Ital J Pediatr. 2019;45:112.

8. Menzies-Gow A, Ying S, Sabroe I, Stubbs VL, Soler D, Williams TJ, et al. Eotaxin (CCL11) and Eotaxin-2 (CCL24) Induce Recruitment of Eosinophils, Basophils, Neutrophils, and Macrophages As Well As Features of Early- and Late-Phase Allergic Reactions Following Cutaneous Injection in Human Atopic and Nonatopic Volunteers. J Immunol. 2002;169:2712–8.

9. Teixeira AL, Gama CS, Rocha NP, Teixeira MM. Revisiting the Role of Eotaxin-1/CCL11 in Psychiatric Disorders. Frontiers Psychiatry. 2018;9:241.

10. Ivanovska M, Abdi Z, Murdjeva M, Macedo D, Maes A, Maes M. CCL-11 or Eotaxin-1: An Immune Marker for Ageing and Accelerated Ageing in Neuro-Psychiatric Disorders. Pharm. 2020;13:230.

11. Otsubo Y, Hashimoto K, Kanbe T, Sumi M, Moriuchi H. Association of cord blood chemokines and other biomarkers with neonatal complications following intrauterine inflammation. Burd I, editor. PLoS ONE. 2017;12:e0175082-12.

12. Zein H, Mohammad K, Leijser LM, Brundler M-A, Kirton A, Esser MJ. Cord Blood Cytokine Levels Correlate With Types of Placental Pathology in Extremely Preterm Infants. Frontiers Pediatrics. 2021;9:607684.

13. Kandasamy J, Roane C, Szalai A, Ambalavanan N. Serum eotaxin-1 is increased in extremely-low-birth-weight infants with bronchopulmonary dysplasia or death. Pediatr Res. 2015;78:498–504.

14. Sellmer A, Henriksen TB, Palmfeldt J, Bech BH, Astono J, Bennike TB, et al. The Patent Ductus Arteriosus in Extremely Preterm Neonates Is More than a Hemodynamic Challenge: New Molecular Insights. Biomol. 2022;12:1179.

15. Cheng Y, Zhu X, Linghu D, Xu Y, Liang J. Serum levels of cytokines in infants treated with conbercept for retinopathy of prematurity. Sci Rep-uk. 2020;10:12695.

16. Sato T, Kusaka S, Shimojo H, Fujikado T. Simultaneous Analyses of Vitreous Levels of 27 Cytokines in Eyes with Retinopathy of Prematurity. Ophthalmology. 2009;116:2165–9.

17. Chybowska AD, Gadd DA, Cheng Y, Bernabeu E, Campbell A, Walker RM, et al. Augmenting clinical risk prediction of cardiovascular disease through protein and epigenetic biomarkers. Medrxiv. 2022;2022.10.21.22281355.

18. Krohn SC, Bonvin P, Proudfoot AEI. CCL18 Exhibits a Regulatory Role through Inhibition of Receptor and Glycosaminoglycan Binding. PLoS ONE. 2013;8:e72321.

19. Chenivesse C, Chang Y, Azzaoui I, Yahia SA, Morales O, Plé C, et al. Pulmonary CCL18 Recruits Human Regulatory T Cells. J Immunol. 2012;189:128–37.

20. Zhong W, Danielsson H, Tebani A, Karlsson MJ, Elfvin A, Hellgren G, et al. Dramatic changes in blood protein levels during the first week of life in extremely preterm infants. Pediatr Res. 2021;89:604–12.

21. Kallankari H, Huusko JM, Kaukola T, Ojaniemi M, Mahlman M, Marttila R, et al. Cerebral Palsy and Polymorphism of the Chemokine CCL18 in Very Preterm Children. Neonatology. 2015;108:124–9.

22. Kallankari H, Kaukola T, Ojaniemi M, Herva R, Perhomaa M, Vuolteenaho R, et al. Chemokine CCL18 predicts intraventricular hemorrhage in very preterm infants. Ann Med. 2010;42:416–25.

23. VanRaemdonck K, Umar S, Palasiewicz K, Volkov S, Volin MV, Arami S, et al. CCL21/CCR7 signaling in macrophages promotes joint inflammation and Th17-mediated osteoclast formation in rheumatoid arthritis. Cell Mol Life Sci. 2020;77:1387–99.

24. Noor S, Wilson EH. Role of C-C chemokine receptor type 7 and its ligands during neuroinflammation. J Neuroinflammation. 2012;9:77.

25. Kingsmore SF, Kennedy N, Halliday HL, Velkinburgh JCV, Zhong S, Gabriel V, et al. Identification of Diagnostic Biomarkers for Infection in Premature Neonates. Mol Cell Proteomics. 2008;7:1863–75.

26. Xia D, Wang S, Liu A, Li L, Zhou P, Xu S. CCL25 Inhibition Alleviates Sepsis-Induced Acute Lung Injury and Inflammation. Infect Drug Resist. 2022;15:3309–21.

27. Yoshie O, Matsushima K. CCR4 and its ligands: from bench to bedside. Int Immunol. 2015;27:11–20.

28. Dogan RE, Long N, Forde E, Dennis K, Kohm AP, Miller SD, et al. CCL22 regulates experimental autoimmune encephalomyelitis by controlling inflammatory macrophage accumulation and effector function. J Leukoc Biol. 2011;89:93–104.

29. Laurikainen H, Vuorela A, Toivonen A, Reinert-Hartwall L, Trontti K, Lindgren M, et al. Elevated serum chemokine CCL22 levels in first-episode psychosis: associations with symptoms, peripheral immune state and in vivo brain glial cell function. Transl Psychiatry. 2020;10:94.

30. Richter JR, Sutton JM, Belizaire RM, Friend LA, Schuster RM, Johannigman TA, et al. Macrophage-Derived Chemokine (CCL22) Is a Novel Mediator of Lung Inflammation Following Hemorrhage and Resuscitation. Shock. 2014;42:525–31.

31. Galaz J, Romero R, Arenas-Hernandez M, Farias-Jofre M, Motomura K, Liu Z, et al. Clarithromycin prevents preterm birth and neonatal mortality by dampening alarmin-induced maternal–fetal inflammation in mice. Bmc Pregnancy Childb. 2022;22:503.

32. Wu X, Sun M, Yang Z, Lu C, Wang Q, Wang H, et al. The Roles of CCR9/CCL25 in Inflammation and Inflammation-Associated Diseases. Front Cell Dev Biol. 2021;9:686548.

33. Abe S, Onoda R, Furushima D, Yamada H, Tamura Y, Sayama K. Detection of CCL25 and the correlation between CCL25, CCL28, IL-7, and TSLP in human breast milk. J Reprod Immunol. 2023;155:103783.

34. Laudanski P, Lemancewicz A, Kuc P, Charkiewicz K, Ramotowska B, Kretowska M, et al. Chemokines Profiling of Patients with Preterm Birth. Mediat Inflamm. 2014;2014:185758.

35. Sanjurjo L, Aran G, Roher N, Valledor AF, Sarrias M. AIM/CD5L: a key protein in the control of immune homeostasis and inflammatory disease. J Leukoc Biol. 2015;98:173–84.

36. Oliveira L, Gomes AP, Santos RF, Cardoso MS, Nóvoa A, Luche H, et al. CD5L constraints acute and systemic inflammation and can be a novel potent therapeutic agent against sepsis. bioRxiv. 2022;2022.03.08.483540.

37. Muk T, Leto A, Brunse A, Stensballe A, Thymann T, Sangild PT, et al. Neonatal prophylactic antibiotics after preterm birth affect plasma proteome and immune development in pigs. Pediatr Res. 2023;1–9.

38. Maehara N, Taniguchi K, Okuno A, Ando H, Hirota A, Li Z, et al. AIM/CD5L attenuates DAMPs in the injured brain and thereby ameliorates ischemic stroke. Cell Rep. 2021;36:109693.

39. Sarrias M-R, Farnós M, Mota R, Sánchez-Barbero F, Ibáñez A, Gimferrer I, et al. CD6 binds to pathogen-associated molecular patterns and protects from LPS-induced septic shock. Proc Natl Acad Sci. 2007;104:11724–9.

40. Català C, Andrés MV, Leyton-Pereira A, Casadó-Llombart S, Moya MS, Gutiérrez-Cózar R, et al. CD6 deficiency impairs early immune response to bacterial sepsis. iScience. 2022;25:105078.

41. Cano-Gamez E, Burnham KL, Goh C, Allcock A, Malick ZH, Overend L, et al. An immune dysfunction score for stratification of patients with acute infection based on whole-blood gene expression. Sci Transl Med. 2022;14:eabq4433–eabq4433.

42. Meyer A, Kofler DM. Failure of a T cell regulator: CD6 contributes to the aggravation of autoimmune inflammation. Cell Mol Immunol. 2019;16:733–4.

43. González-Cabrero J, Wise CJ, Latchman Y, Freeman GJ, Sharpe AH, Reiser H. CD48-deficient mice have a pronounced defect in CD4+ T cell activation. Proc Natl Acad Sci. 1999;96:1019–23.

44. Boles NC, Lin KK, Lukov GL, Bowman TV, Baldridge MT, Goodell MA. CD48 on hematopoietic progenitors regulates stem cells and suppresses tumor formation. Blood. 2011;118:80–7.

45. McArdel SL, Terhorst C, Sharpe AH. Roles of CD48 in regulating immunity and tolerance. Clin Immunol. 2016;164:10–20.

46. Mirchandani AS, Jenkins SJ, Bain CC, Sanchez-Garcia MA, Lawson H, Coelho P, et al. Hypoxia shapes the immune landscape in lung injury and promotes the persistence of inflammation. Nat Immunol. 2022;23:927–39.

47. Zhou J, Zhao L, Xiao Y, Xie S, Long Y, Wei Y, et al. The Expression of Cytokine Profiles and Related Receptors in Idiopathic Inflammatory Myopathies. Front Pharmacol. 2022;13:852055.

48. Bhatti G, Romero R, Gomez-Lopez N, Pique-Regi R, Pacora P, Jung E, et al. The amniotic fluid cell-free transcriptome in spontaneous preterm labor. Sci Rep. 2021;11:13481.

49. Costa D, Castelo R. Umbilical cord gene expression reveals the molecular architecture of the fetal inflammatory response in extremely preterm newborns. Pediatr Res. 2016;79:473–81.

50. Fabriek BO, Bruggen R van, Deng DM, Ligtenberg AJM, Nazmi K, Schornagel K, et al. The macrophage scavenger receptor CD163 functions as an innate immune sensor for bacteria. Blood. 2009;113:887–92.

51. Etzerodt A, Moestrup SK. CD163 and Inflammation: Biological, Diagnostic, and Therapeutic Aspects. Antioxid Redox Sign. 2013;18:2352–63.

52. Gustafsson AM, Fransson E, Dubicke A, Hjelmstedt AK, Ekman‐Ordeberg G, Silfverdal S, et al. Low levels of anti‐secretory factor in placenta are associated with preterm birth and inflammation. Acta Obstet Gyn Scan. 2018;97:349–56.

53. Olaloye OO, Liu P, Toothaker JM, McCourt BT, McCourt CC, Xiao J, et al. CD16+CD163+ monocytes traffic to sites of inflammation during necrotizing enterocolitis in premature infants. J Exp Med. 2021;218:e20200344.

54. Groselj-Grenc M, Ihan A, Derganc M. Neutrophil and Monocyte CD64 and CD163 Expression in Critically Ill Neonates and Children with Sepsis: Comparison of Fluorescence Intensities and Calculated Indexes. Mediat Inflamm. 2008;2008:202646.

55. Waleh N, Seidner S, McCurnin D, Giavedoni L, Hodara V, Goelz S, et al. Anatomic Closure of the Premature Patent Ductus Arteriosus: The Role of CD14+/CD163+ Mononuclear Cells and VEGF in Neointimal Mound Formation. Pediatr Res. 2011;70:332–8.

56. Schlitzer A, McGovern N, Ginhoux F. Dendritic cells and monocyte-derived cells: Two complementary and integrated functional systems. Semin Cell Dev Biol. 2015;41:9–22.

57. Kämmerer U, Eggert AO, Kapp M, McLellan AD, Geijtenbeek TBH, Dietl J, et al. Unique Appearance of Proliferating Antigen-Presenting Cells Expressing DC-SIGN (CD209) in the Decidua of Early Human Pregnancy. Am J Pathol. 2003;162:887–96.

58. Gomez-Lopez N, Garcia-Flores V, Chin PY, Groome HM, Bijland MT, Diener KR, et al. Macrophages exert homeostatic actions in pregnancy to protect against preterm birth and fetal inflammatory injury. Jci Insight. 2021;6:e146089.

59. Marzaioli V, Canavan M, Floudas A, Flynn K, Mullan R, Veale DJ, et al. CD209/CD14+ Dendritic Cells Characterization in Rheumatoid and Psoriatic Arthritis Patients: Activation, Synovial Infiltration, and Therapeutic Targeting. Front Immunol. 2022;12:722349.

60. Zilow EP, Hauck W, Linderkamp O, Zilow G. Alternative Pathway Activation of the Complement System in Preterm Infants with Early Onset Infection. Pediatr Res. 1997;41:334–9.

61. Sullivan G, Galdi P, Blesa-Cábez M, Borbye-Lorenzen N, Stoye DQ, Lamb GJ, et al. Interleukin-8 dysregulation is implicated in brain dysmaturation following preterm birth. Brain Behav Immun. 2020;90:311–8.

62. Sjöholm AG, Jönsson G, Braconier JH, Sturfelt G, Truedsson L. Complement deficiency and disease: An update. Mol Immunol. 2006;43:78–85.

63. Lassiter HA, Walz BM, Wilson JL, Jung E, Calisi CR, Goldsmith LJ, et al. The Administration of Complement Component C9 Enhances the Survival of Neonatal Rats with Escherichia coli Sepsis. Pediatr Res. 1997;42:128–36.

64. Adinolfi M, Beck SE. Human complement C7 and C9 in fetal and newborn sera. Arch Dis Child. 1975;50:562.

65. Ballow M, Fang F, Good RA, Day NK. Developmental aspects of complement components in the newborn: The presence of complement components and C3 proactivator (properdin factor B) in human colostrum. Clin Exp Immunol. 1974;18:257–66.

66. Beernink RHJ, Schuitemaker JHN, Zwertbroek EF, Scherjon SA, Cremers TIFH. Early pregnancy biomarker discovery study for spontaneous preterm birth. Placenta. 2023;139:112–9.

67. Segura-Cervantes E, Mancilla-Ramirez J, Zurita L, Paredes Y, Arredondo JL, Galindo-Sevilla N. Blood SC5b-9 complement levels increase at parturition during term and preterm labor. J Reprod Immunol. 2015;109:24–30.

68. Schultz SJ, Aly H, Hasanen BM, Khashaba MT, Lear SC, Bendon RW, et al. Complement component 9 activation, consumption, and neuronal deposition in the post-hypoxic–ischemic central nervous system of human newborn infants. Neurosci Lett. 2005;378:1–6.

69. Aly H, Khashaba M, Nada A, Hasanen B, McCarter R, Schultz S, et al. The Role of Complement in Neurodevelopmental Impairment following Neonatal Hypoxic-Ischemic Encephalopathy. Am J Perinatol. 2009;26:659–65.

70. Shimoda Y, Watanabe K. Contactins: Emerging key roles in the development and function of the nervous system. Cell Adhes Migr. 2009;3:64–70.

71. Chatterjee M, Schild D, Teunissen CE. Contactins in the central nervous system: role in health and disease. Neural Regen Res. 2019;14:206–16.

72. Cottrell CE, Bir N, Varga E, Alvarez CE, Bouyain S, Zernzach R, et al. Contactin 4 as an autism susceptibility locus. Autism Res. 2011;4:189–99.

73. Roohi J, Montagna C, Tegay DH, Palmer LE, DeVincent C, Pomeroy JC, et al. Disruption of contactin 4 in three subjects with autism spectrum disorder. J Méd Genet. 2009;46:176.

74. Oguro-Ando A, Bamford RA, Sital W, Sprengers JJ, Zuko A, Matser JM, et al. Cntn4, a risk gene for neuropsychiatric disorders, modulates hippocampal synaptic plasticity and behavior. Transl Psychiatry. 2021;11:106.

75. DuClos TW. Function of C-reactive protein. Ann Med. 2000;32:274–8.

76. Sullivan G, Galdi P, Borbye-Lorenzen N, Stoye DQ, Lamb GJ, Evans MJ, et al. Preterm Birth Is Associated With Immune Dysregulation Which Persists in Infants Exposed to Histologic Chorioamnionitis. Front Immunol. 2021;12:722489.

77. Skogstrand K, Hougaard DM, Schendel DE, Bent N-P, Sværke C, Thorsen P. Association of Preterm Birth With Sustained Postnatal Inflammatory Response. Obstetrics Gynecol. 2008;111:1118–28.

78. Leviton A, Allred EN, Fichorova RN, O’Shea TM, Fordham LA, Kuban KKC, et al. Circulating biomarkers in extremely preterm infants associated with ultrasound indicators of brain damage. Eur J Paediatr Neuro. 2018;22:440–50.

79. Leviton A, Joseph RM, Fichorova RN, Allred EN, Taylor HG, O’Shea TM, et al. Executive Dysfunction Early Postnatal Biomarkers among Children Born Extremely Preterm. J Neuroimmune Pharm. 2019;14:188–99.

80. Allred EN, Dammann O, Fichorova RN, Hooper SR, Hunter SJ, Joseph RM, et al. Systemic Inflammation during the First Postnatal Month and the Risk of Attention Deficit Hyperactivity Disorder Characteristics among 10 year-old Children Born Extremely Preterm. J Neuroimmune Pharm. 2017;12:531–43.

81. Kuban KCK, O’Shea TM, Allred EN, Paneth N, Hirtz D, Fichorova RN, et al. Systemic Inflammation and Cerebral Palsy Risk in Extremely Preterm Infants. J Child Neurol. 2013;29:1692–8.

82. Conole ELS, Stevenson AJ, Maniega SM, Harris SE, Green C, Hernández M del CV, et al. DNA Methylation and Protein Markers of Chronic Inflammation and Their Associations With Brain and Cognitive Aging. Neurology. 2021;97:e2340–52.

83. Green C, Shen X, Stevenson AJ, Conole ELS, Harris MA, Barbu MC, et al. Structural brain correlates of serum and epigenetic markers of inflammation in major depressive disorder. Brain Behav Immun. 2021;92:39–48.

84. Barker ED, Cecil CAM, Walton E, Houtepen LC, O’Connor TG, Danese A, et al. Inflammation-related epigenetic risk and child and adolescent mental health: A prospective study from pregnancy to middle adolescence. Dev Psychopathol. 2018;30:1145–56.

85. Edmondson-Stait AJ, Shen X, Adams MJ, Barbu MC, Jones HJ, Miron VE, et al. Early-life inflammatory markers and subsequent psychotic and depressive episodes between 10 to 28 years of age. Brain Behav Immun - Heal. 2022;26:100528.

86. Conole ELS, Vaher K, Blesa-Cábez M, Sullivan G, Stevenson AJ, Hall J, et al. Immuno-epigenetic signature derived in saliva associates with the encephalopathy of prematurity and perinatal inflammatory disorders. Brain Behav Immun. 2023;110:322–38.

87. Yeh J-H, Sidhu SS, Chan AC. Regulation of a Late Phase of T Cell Polarity and Effector Functions by Crtam. Cell. 2008;132:846–59.

88. Cortez VS, Cervantes-Barragan L, Song C, Gilfillan S, McDonald KG, Tussiwand R, et al. CRTAM controls residency of gut CD4+CD8+ T cells in the steady state and maintenance of gut CD4+ Th17 during parasitic infection. J Exp Med. 2014;211:623–33.

89. Cervantes-Barragan L, Cortez VS, Wang Q, McDonald KG, Chai JN, Luccia BD, et al. CRTAM Protects Against Intestinal Dysbiosis During Pathogenic Parasitic Infection by Enabling Th17 Maturation. Front Immunol. 2019;10:1423.

90. Perez-Lopez A, Nuccio S-P, Ushach I, Edwards RA, Pahu R, Silva S, et al. CRTAM Shapes the Gut Microbiota and Enhances the Severity of Infection. J Immunol. 2019;203:532–43.

91. Hardison JL, Wrightsman RA, Carpenter PM, Lane TE, Manning JE. The Chemokines CXCL9 and CXCL10 Promote a Protective Immune Response but Do Not Contribute to Cardiac Inflammation following Infection with Trypanosoma cruzi. Infect Immun. 2006;74:125–34.

92. Fried M, Kurtis JD, Swihart B, Pond-Tor S, Barry A, Sidibe Y, et al. Systemic Inflammatory Response to Malaria During Pregnancy Is Associated With Pregnancy Loss and Preterm Delivery. Clin Infect Dis. 2017;65:1729–35.

93. Ronzoni S, Steckle V, D’Souza R, Murphy KE, Lye S, Shynlova O. Cytokine Changes in Maternal Peripheral Blood Correlate With Time-to-Delivery in Pregnancies Complicated by Premature Prelabor Rupture of the Membranes. Reprod Sci. 2019;26:1266–76.

94. Schettini JA de C, Gomes TV, Barreto AKS, Júnior CD da S, Matta M da, Coutinho ICN, et al. High Levels of CXCL8 and Low Levels of CXCL9 and CXCL10 in Women with Maternal RhD Alloimmunization. Front Immunol. 2017;8:700.

95. Kim MJ, Romero R, Kim CJ, Tarca AL, Chhauy S, LaJeunesse C, et al. Villitis of Unknown Etiology Is Associated with a Distinct Pattern of Chemokine Up-Regulation in the Feto-Maternal and Placental Compartments: Implications for Conjoint Maternal Allograft Rejection and Maternal Anti-Fetal Graft-versus-Host Disease. J Immunol. 2009;182:3919–27.

96. McKimmie C, Michlmayr D. Role of CXCL10 in central nervous system inflammation. Int J Interf Cytokine Mediat Res. 2014;1.

97. Zhang Y, Sun B, Hu M, Lou Y, Lu J, Zhang X, et al. CXCL9 as a Prognostic Inflammatory Marker in Early-Stage Lung Adenocarcinoma Patients. Front Oncol. 2020;10:1049.

98. Kameda M, Otsuka M, Chiba H, Kuronuma K, Hasegawa T, Takahashi H, et al. CXCL9, CXCL10, and CXCL11; biomarkers of pulmonary inflammation associated with autoimmunity in patients with collagen vascular diseases–associated interstitial lung disease and interstitial pneumonia with autoimmune features. PLoS ONE. 2020;15:e0241719.

99. Vazirinejad R, Ahmadi Z, Arababadi MK, Hassanshahi G, Kennedy D. The Biological Functions, Structure and Sources of CXCL10 and Its Outstanding Part in the Pathophysiology of Multiple Sclerosis. Neuroimmunomodulat. 2014;21:322–30.

100. Romero R, Chaemsaithong P, Chaiyasit N, Docheva N, Dong Z, Kim CJ, et al. CXCL10 and IL‐6: Markers of two different forms of intra‐amniotic inflammation in preterm labor. Am J Reprod Immunol. 2017;78:e12685.

101. Aminzadeh F, Ghorashi Z, Nabati S, Ghasemshirazi M, Arababadi MK, Shamsizadeh A, et al. Differential Expression of CXC Chemokines CXCL10 and CXCL12 in Term and Pre‐term Neonates and Their Mothers. Am J Reprod Immunol. 2012;68:338–44.

102. Satrom KM, Ennis K, Sweis BM, Matveeva TM, Chen J, Hanson L, et al. Neonatal hyperglycemia induces CXCL10/CXCR3 signaling and microglial activation and impairs long-term synaptogenesis in the hippocampus and alters behavior in rats. J Neuroinflamm. 2018;15:82.

103. Wisgrill L, Muck M, Wessely I, Berger A, Spittler A, Förster-Waldl E, et al. Endothelial cells of extremely premature infants display impaired immune response after proinflammatory stimulation. Pediatr Res. 2018;83:128–34.

104. Che X, Hornig M, Bresnahan M, Stoltenberg C, Magnus P, Surén P, et al. Maternal mid-gestational and child cord blood immune signatures are strongly associated with offspring risk of ASD. Mol Psychiatr. 2022;27:1527–41.

105. Fitzgerald AA, Weiner LM. The role of fibroblast activation protein in health and malignancy. Cancer Metastasis Rev. 2020;39:783–803.

106. Ohmaru-Nakanishi T, Asanoma K, Fujikawa M, Fujita Y, Yagi H, Onoyama I, et al. Fibrosis in Preeclamptic Placentas Is Associated with Stromal Fibroblasts Activated by the Transforming Growth Factor-β1 Signaling Pathway. Am J Pathol. 2018;188:683–95.

107. Li X, Zhang B, Ding W, Jia X, Han Z, Zhang L, et al. Serum Proteomic Signatures in Umbilical Cord Blood of Preterm Neonates Delivered by Women with Gestational Diabetes. Diabetes, Metab Syndr Obes. 2023;16:1525–39.

108. Mkaddem SB, Benhamou M, Monteiro RC. Understanding Fc Receptor Involvement in Inflammatory Diseases: From Mechanisms to New Therapeutic Tools. Front Immunol. 2019;10:811.

109. McKinney C, Broen JCA, Vonk MC, Beretta L, Hesselstrand R, Hunzelmann N, et al. Evidence that deletion at FCGR3B is a risk factor for systemic sclerosis. Genes Immun. 2012;13:458–60.

110. Graf SW, Lester S, Nossent JC, Hill CL, Proudman SM, Lee A, et al. Low copy number of the FCGR3B gene and rheumatoid arthritis: a case-control study and meta-analysis. Arthritis Res Ther. 2012;14:R28.

111. Asano K, Matsumoto T, Umeno J, Hirano A, Esaki M, Hosono N, et al. Impact of Allele Copy Number of Polymorphisms in FCGR3A and FCGR3B Genes on Susceptibility to Ulcerative Colitis. Inflamm Bowel Dis. 2013;19:2061–8.

112. Aitman TJ, Dong R, Vyse TJ, Norsworthy PJ, Johnson MD, Smith J, et al. Copy number polymorphism in Fcgr3 predisposes to glomerulonephritis in rats and humans. Nature. 2006;439:851–5.

113. Adu B, Dodoo D, Adukpo S, Hedley PL, Arthur FKN, Gerds TA, et al. Fc Gamma Receptor IIIB (FcγRIIIB) Polymorphisms Are Associated with Clinical Malaria in Ghanaian Children. PLoS ONE. 2012;7:e46197.

114. Jackson TA, Haga CL, Ehrhardt GRA, Davis RS, Cooper MD. FcR-Like 2 Inhibition of B Cell Receptor-Mediated Activation of B Cells. J Immunol. 2010;185:7405–12.

115. Masuda K, Mori H, Ohara O, Nakayama M, Wang J-Y, Burrows PD. Defining the immunological phenotype of Fc receptor-like B (FCRLB) deficient mice: Confounding role of the inhibitory FcγRIIb. Cell Immunol. 2010;266:24–31.

116. Comabella M, Cantó E, Nurtdinov R, Río J, Villar LM, Picón C, et al. MRI phenotypes with high neurodegeneration are associated with peripheral blood B-cell changes. Hum Mol Genet. 2016;25:308–16.

117. Khanzadeh A, Habibagahi Z, Hosseini A, Amirghofran Z. Investigation of the human FCRL1, 2, and 4 gene expressions in patients with rheumatoid arthritis. Rheumatol Int. 2016;36:1149–56.

118. Shea LK, Honjo K, Redden DT, Tabengwa E, Li R, Li F-J, et al. Fc receptor-like 2 (FCRL2) is a novel marker of low-risk CLL and refines prognostication based on IGHV mutation status. Blood Cancer J. 2019;9:47.

119. Olvera‐Rojas M, Plaza‐Florido A, Solis‐Urra P, Rodriguez‐Ayllon M, Toval A, Esteban‐Cornejo I, et al. Association of muscular strength and targeted proteomics involved in brain health in children with overweight/obesity. Scand J Med Sci Sports. 2023;33:1738–51.

120. Tezze C, Romanello V, Sandri M. FGF21 as Modulator of Metabolism in Health and Disease. Front Physiol. 2019;10:419.

121. Guasti L, Silvennoinen S, Bulstrode NW, Ferretti P, Sankilampi U, Dunkel L. Elevated FGF21 Leads to Attenuated Postnatal Linear Growth in Preterm Infants Through GH Resistance in Chondrocytes. J Clin Endocrinol Metab. 2014;99:E2198–206.

122. Spencer R, Maksym K, Hecher K, Maršál K, Figueras F, Ambler G, et al. Ultrasound and biochemical predictors of pregnancy outcome at diagnosis of early-onset fetal growth restriction. Medrxiv. 2023;2023.01.27.23285087.

123. Breit SN, Johnen H, Cook AD, Tsai VWW, Mohammad MG, Kuffner T, et al. The TGF-β superfamily cytokine, MIC-1/GDF15: A pleotrophic cytokine with roles in inflammation, cancer and metabolism. Growth Factors. 2011;29:187–95.

124. Wischhusen J, Melero I, Fridman WH. Growth/Differentiation Factor-15 (GDF-15): From Biomarker to Novel Targetable Immune Checkpoint. Front Immunol. 2020;11:951.

125. Almudares F, Hagan J, Chen X, Devaraj S, Moorthy B, Lingappan K. Growth and differentiation factor 15 (GDF15) levels predict adverse respiratory outcomes in premature neonates. Pediatr Pulmonol. 2023;58:271–8.

126. Kinoshita M, Yatsuga S, Iwata O, Okamura H, Morisaki T, Iwata S, et al. Temporal changes and control variables of growth differentiation factor 15 levels during the first week of life in hospitalised newborn infants. Mitochondrion. 2021;61:25–30.

127. Li H, Tang D, Chen J, Hu Y, Cai X, Zhang P. The Clinical Value of GDF15 and Its Prospective Mechanism in Sepsis. Front Immunol. 2021;12:710977.

128. Wertaschnigg D, Rolnik DL, Nie G, Teoh SSY, Syngelaki A, Costa F da S, et al. Second‐ and third‐trimester serum levels of growth‐differentiation factor‐15 in prediction of pre‐eclampsia. Ultrasound Obst Gyn. 2020;56:879–84.

129. Yatsuga S, Fujita Y, Ishii A, Fukumoto Y, Arahata H, Kakuma T, et al. Growth differentiation factor 15 as a useful biomarker for mitochondrial disorders. Ann Neurol. 2015;78:814–23.

130. Wu P-F, Zhang X-H, Zhou P, Yin R, Zhou X-T, Zhang W. Growth Differentiation Factor 15 Is Associated With Alzheimer’s Disease Risk. Frontiers Genetics. 2021;12:700371.

131. Al-Mudares F, Reddick S, Ren J, Venkatesh A, Zhao C, Lingappan K. Role of Growth Differentiation Factor 15 in Lung Disease and Senescence: Potential Role Across the Lifespan. Frontiers Medicine. 2020;7:594137.

132. Wan Y, Fu J. GDF15 as a key disease target and biomarker: linking chronic lung diseases and ageing. Mol Cell Biochem. 2023;1–14.

133. Zhang Y, Jiang W, Wang L, Lingappan K. Sex-specific differences in the modulation of Growth Differentiation Factor 15 (GDF15) by hyperoxia in vivo and in vitro: Role of Hif-1α. Toxicol Appl Pharm. 2017;332:8–14.

134. Olsson KW, Larsson A, Jonzon A, Sindelar R. Exploration of potential biochemical markers for persistence of patent ductus arteriosus in preterm infants at 22–27 weeks’ gestation. Pediatr Res. 2019;86:333–8.

135. Lohani O, Colvin KL, Yeager ME. Biomarkers for pediatric pulmonary arterial hypertension: challenges and recommendations. Paediatr Respir Rev. 2015;16:225–31.

136. Butler AA, Roith DL. Control of growth by the somatropic axis: growth hormone and the insulin-like growth factors have related and independent roles. Annual Review of Physiology. 2001;63:141–64.

137. Zaghlool SB, Sharma S, Molnar M, Matías-García PR, Elhadad MA, Waldenberger M, et al. Revealing the role of the human blood plasma proteome in obesity using genetic drivers. Nat Commun. 2021;12:1279.

138. Schreiner F, Stutte S, Bartmann P, Gohlke B, Woelfle J. Association of the Growth Hormone Receptor d3-Variant and Catch-up Growth of Preterm Infants with Birth Weight of Less Than 1500 Grams. J Clin Endocrinol Metab. 2007;92:4489–93.

139. deBie HMA, Oostrom KJ, Delemarre-vandeWaal HA. Brain Development, Intelligence and Cognitive Outcome in Children Born Small for Gestational Age. Horm Res Paediat. 2010;73:6–14.

140. Dourson AJ, Ford ZK, Green KJ, McCrossan CE, Hofmann MC, Hudgins RC, et al. Early Life Nociception is Influenced by Peripheral Growth Hormone Signaling. J Neurosci. 2021;41:4410–27.

141. Masternak MM, Bartke A. Growth hormone, inflammation and aging. Pathobiol Aging Age-Relat Dis. 2012;2:17293.

142. Huang Z, Xiao L, Xiao Y, Chen C. The Modulatory Role of Growth Hormone in Inflammation and Macrophage Activation. Endocrinology. 2022;163.

143. Andersson TR, Bangstad H, Larsen ML. Heparin Cofactor II, Antithrombin and Protein C in Plasma from Term and Preterm Infants. Acta Pædiatrica. 1988;77:485–8.

144. Kalle M, Papareddy P, Kasetty G, Plas MJA van der, Mörgelin M, Malmsten M, et al. A Peptide of Heparin Cofactor II Inhibits Endotoxin-Mediated Shock and Invasive Pseudomonas aeruginosa Infection. PLoS ONE. 2014;9:e102577.

145. Kalle M, Papareddy P, Kasetty G, Tollefsen DM, Malmsten M, Mörgelin M, et al. Proteolytic Activation Transforms Heparin Cofactor II into a Host Defense Molecule. J Immunol. 2013;190:6303–10.

146. Tollefsen DM. Heparin Cofactor II Modulates the Response to Vascular Injury. Arter, Thromb, Vasc Biol. 2007;27:454–60.

147. Kurahashi K, Inoue S, Yoshida S, Ikeda Y, Morimoto K, Uemoto R, et al. The Role of Heparin Cofactor Ⅱ in the Regulation of Insulin Sensitivity and Maintenance of Glucose Homeostasis in Humans and Mice. J Atheroscler Thromb. 2017;24:37739.

148. Molnarfi N, Benkhoucha M, Funakoshi H, Nakamura T, Lalive PH. Hepatocyte growth factor: A regulator of inflammation and autoimmunity. Autoimmun Rev. 2015;14:293–303.

149. Jimenez-Gomez G, Benavente-Fernandez I, Lubian-Lopez SP, Matias-Vega M, Lechuga-Campoy JL, Saez-Benito A, et al. Hepatocyte growth factor as an indicator of neonatal maturity. J Pediatr Endocrinol Metab. 2013;26:709–14.

150. Bränn E, Fransson E, White RA, Papadopoulos FC, Edvinsson Å, Kamali‐Moghaddam M, et al. Inflammatory markers in women with postpartum depressive symptoms. J Neurosci Res. 2020;98:1309–21.

151. Lassus P, Heikkilä P, Andersson LC, Boguslawski K von, Andersson S. Lower concentration of pulmonary hepatocyte growth factor is associated with more severe lung disease in preterm infants. J Pediatr. 2003;143:199–202.

152. Ohki Y, Mayuzumi H, Tokuyama K, Yoshizawa Y, Arakawa H, Mochizuki H, et al. Hepatocyte Growth Factor Treatment Improves Alveolarization in a Newborn Murine Model of Bronchopulmonary Dysplasia. Neonatology. 2009;95:332–8.

153. Rajaram S, Baylink DJ, Mohan S. Insulin-Like Growth Factor-Binding Proteins in Serum and Other Biological Fluids: Regulation and Functions. Endocr Rev. 1997;18:801–31.

154. Qiu Q, Bell M, Lu X, Yan X, Rodger M, Walker M, et al. Significance of IGFBP-4 in the Development of Fetal Growth Restriction. J Clin Endocrinol Metab. 2012;97:E1429–39.

155. Price WA, Moats-Staats BM, Stiles AD. Pro- and Anti-inflammatory Cytokines Regulate Insulin-like Growth Factor  Binding Protein Production by Fetal Rat Lung Fibroblasts. Am J Respir Cell Mol Biol. 2002;26:283–9.

156. DiToro D, Harbour SN, Bando JK, Benavides G, Witte S, Laufer VA, et al. Insulin-Like Growth Factors Are Key Regulators of T Helper 17 Regulatory T Cell Balance in Autoimmunity. Immunity. 2020;52:650-667.e10.

157. Alessio N, Squillaro T, Bernardo GD, Galano G, Rosa RD, Melone MA, et al. Increase of circulating IGFBP-4 following genotoxic stress and its implication for senescence. eLife. 2020;9:e54523.

158. Ivetic A, Green HLH, Hart SJ. L-selectin: A Major Regulator of Leukocyte Adhesion, Migration and Signaling. Front Immunol. 2019;10:1068.

159. Bose CL, Dammann CEL, Laughon MM. Bronchopulmonary dysplasia and inflammatory biomarkers in the premature neonate. Archives Dis Child - Fetal Neonatal Ed. 2008;93:F455.

160. Hashimoto M, Nishida A, Minakami H, Takashima Y, Kato M, Okada Y, et al. Decreased Expression of L-Selectin on Peripheral Blood Polymorphonuclear Leukocytes in Neonates with Severe Asphyxia. Neonatology. 2002;81:95–8.

161. Lonnerdal B, Iyer S. Lactoferrin: Molecular structure and biological function. Annu Rev Nutr. 1995;93–110.

162. Ochoa TJ, Sizonenko SV. Lactoferrin and prematurity: a promising milk protein? Biochem Cell Biol. 2017;95:22–30.

163. Pammi M, Suresh G. Enteral lactoferrin supplementation for prevention of sepsis and necrotizing enterocolitis in preterm infants. Cochrane Db Syst Rev. 2020;3:CD007137.

164. Cornish J, Callon KE, Naot D, Palmano KP, Banovic T, Bava U, et al. Lactoferrin Is a Potent Regulator of Bone Cell Activity and Increases Bone Formation in Vivo. Endocrinology. 2004;145:4366–74.

165. Loffek S, Schilling O, Franzke C-W. Biological role of matrix metalloproteinases: a critical balance. Eur Respir J. 2010;38:191–208.

166. Reinhard SM, Razak K, Ethell IM. A delicate balance: role of MMP-9 in brain development and pathophysiology of neurodevelopmental disorders. Front Cell Neurosci. 2015;9:280.

167. Matoba N, Yu Y, Mestan K, Pearson C, Ortiz K, Porta N, et al. Differential Patterns of 27 Cord Blood Immune Biomarkers Across Gestational Age. Pediatrics. 2009;123:1320–8.

168. Boardman JP, Ireland G, Sullivan G, Pataky R, Fleiss B, Gressens P, et al. The Cerebrospinal Fluid Inflammatory Response to Preterm Birth. Front Physiol. 2018;9:1299.

169. Rathi S, Jalali S, Patnaik S, Shahulhameed S, Musada GR, Balakrishnan D, et al. Abnormal Complement Activation and Inflammation in the Pathogenesis of Retinopathy of Prematurity. Front Immunol. 2017;8:1868.

170. Morales DM, Townsend RR, Malone JP, Ewersmann CA, Macy EM, Inder TE, et al. Alterations in Protein Regulators of Neurodevelopment in the Cerebrospinal Fluid of Infants with Posthemorrhagic Hydrocephalus of Prematurity. Mol Cell Proteom. 2012;11:M111.011973.

171. Course CW, Lewis PA, Kotecha SJ, Cousins M, Hart K, Watkins WJ, et al. Characterizing the urinary proteome of prematurity-associated lung disease in school-aged children. Respir Res. 2023;24:191.

172. Kiss JZ, Müller D. Contribution of the Neural Cell Adhesion Molecule to Neuronal and Synaptic Plasticity. Rev Neurosci. 2001;12:297–310.

173. Limbrick DD, Morales DM, Shannon CN, Wellons JC, Kulkarni AV, Alvey JS, et al. Cerebrospinal fluid NCAM-1 concentration is associated with neurodevelopmental outcome in post-hemorrhagic hydrocephalus of prematurity. PLoS ONE. 2021;16:e0247749.

174. Eve M, Gandawijaya J, Yang L, Oguro-Ando A. Neuronal Cell Adhesion Molecules May Mediate Neuroinflammation in Autism Spectrum Disorder. Frontiers Psychiatry. 2022;13:842755.

175. Jesudas BR, Nandeesha H, Menon V, Allimuthu P. Relationship of elevated neural cell adhesion molecule 1 with interleukin-10 and disease severity in bipolar disorder. Asian J Psychiatry. 2020;47:101849.

176. Shiwaku H, Katayama S, Kondo K, Nakano Y, Tanaka H, Yoshioka Y, et al. Autoantibodies against NCAM1 from patients with schizophrenia cause schizophrenia-related behavior and changes in synapses in mice. Cell Rep Med. 2022;3:100597.

177. Sandi C. Stress, cognitive impairment and cell adhesion molecules. Nature Reviews Neuroscience. 2004;5:917–14.

178. Wang P, Pan J, Tian X, Dong X, Ju W, Wang Y, et al. Transcriptomics‐determined chemokine‐cytokine pathway presents a common pathogenic mechanism in pregnancy loss and spontaneous preterm birth. Am J Reprod Immunol. 2021;86:e13398.

179. Brockway HM, Kallapur SG, Buhimschi IA, Buhimschi CS, Ackerman WE, Muglia LJ, et al. Unique transcriptomic landscapes identified in idiopathic spontaneous and infection related preterm births compared to normal term births. PLoS ONE. 2019;14:e0225062.

180. Starodubtseva NL, Kononikhin AS, Bugrova AE, Chagovets V, Indeykina M, Krokhina KN, et al. Investigation of urine proteome of preterm newborns with respiratory pathologies. J Proteom. 2016;149:31–7.

181. Kaetzel CS. Polymeric Ig receptor: Defender of the fort or Trojan Horse? Curr Biol. 2001;11:R35–8.

182. Wei H, Wang J-Y. Role of Polymeric Immunoglobulin Receptor in IgA and IgM Transcytosis. Int J Mol Sci. 2021;22:2284.

183. Johansen F-E, Kaetzel CS. Regulation of the polymeric immunoglobulin receptor and IgA transport: new advances in environmental factors that stimulate pIgR expression and its role in mucosal immunity. Mucosal Immunol. 2011;4:598–602.

184. Xu C, Su X, Chen Y, Xu Y, Wang Z, Mo X. Proteomics analysis of plasma protein changes in patent ductus arteriosus patients. Ital J Pediatr. 2020;46:64.

185. Wang S, Song R, Wang Z, Jing Z, Wang S, Ma J. S100A8/A9 in Inflammation. Front Immunol. 2018;9:1298.

186. Lee JE, Park KH, Kim HJ, Kim YM, Choi J-W, Shin S, et al. Proteomic identification of novel plasma biomarkers associated with spontaneous preterm birth in women with preterm labor without infection/inflammation. PLoS ONE. 2021;16:e0259265.

187. Willers M, Ulas T, Völlger L, Vogl T, Heinemann AS, Pirr S, et al. S100A8 and S100A9 Are Important for Postnatal Development of Gut Microbiota and Immune System in Mice and Infants. Gastroenterology. 2020;159:2130-2145.e5.

188. Yue R, Shen B, Morrison SJ. Clec11a/osteolectin is an osteogenic growth factor that promotes the maintenance of the adult skeleton. eLife. 2016;5:e18782.

189. Brown GD, Willment JA, Whitehead L. C-type lectins in immunity and homeostasis. Nat Rev Immunol. 2018;18:374–89.

190. Wagner BD, Babinec AE, Carpenter C, Gonzalez S, O’Brien G, Rollock K, et al. Proteomic Profiles Associated with Early Echocardiogram Evidence of Pulmonary Vascular Disease in Preterm Infants. Am J Respir Crit Care Med. 2018;197:394–7.

191. Mohammed A, Okwor I, Shan L, Onyilagha C, Uzonna JE, Gounni AS. Semaphorin 3E Regulates the Response of Macrophages to Lipopolysaccharide-Induced Systemic Inflammation. J Immunol. 2020;204:128–36.

192. Mata A, Gil V, Pérez-Clausell J, Dasilva M, González-Calixto MC, Soriano E, et al. New functions of Semaphorin 3E and its receptor PlexinD1 during developing and adult hippocampal formation. Sci Rep. 2018;8:1381.

193. Sun Y, Liu C, Wang Z, Meng SS, Burnim SB, SanGiovanni JP, et al. RORα modulates semaphorin 3E transcription and neurovascular interaction in pathological retinal angiogenesis. FASEB J. 2017;31:4492–502.

194. Rivera JC, Holm M, Austeng D, Morken TS, Zhou T (Ellen), Beaudry-Richard A, et al. Retinopathy of prematurity: inflammation, choroidal degeneration, and novel promising therapeutic strategies. J Neuroinflamm. 2017;14:165.

195. Jiao B, Liu S, Tan X, Lu P, Wang D, Xu H. Class-3 semaphorins: Potent multifunctional modulators for angiogenesis-associated diseases. Biomed Pharmacother. 2021;137:111329.

196. Kermarrec L, Eissa N, Wang H, Kapoor K, Diarra A, Gounni AS, et al. Semaphorin‐3E attenuates intestinal inflammation through the regulation of the communication between splenic CD11C+ and CD4+CD25− T‐cells. Br J Pharmacol. 2019;176:1235–50.

197. Bergman JEH, Janssen N, Hoefsloot LH, Jongmans MCJ, Hofstra RMW, Ravenswaaij-Arts CMA van. CHD7 mutations and CHARGE syndrome: the clinical implications of an expanding phenotype. J Med Genet. 2011;48:334.

198. Chang Y-C, Olson J, Louie A, Crocker PR, Varki A, Nizet V. Role of macrophage sialoadhesin in host defense against the sialylated pathogen group B Streptococcus. J Mol Med. 2014;92:951–9.

199. Herzog S, Fragkou PC, Arneth BM, Mkhlof S, Skevaki C. Myeloid CD169/Siglec1: An immunoregulatory biomarker in viral disease. Front Med. 2022;9:979373.

200. Oliveira JJ, Karrar S, Rainbow DB, Pinder CL, Clarke P, García AR, et al. The plasma biomarker soluble SIGLEC-1 is associated with the type I interferon transcriptional signature, ethnic background and renal disease in systemic lupus erythematosus. Arthritis Res Ther. 2018;20:152.

201. Ostendorf L, Dittert P, Biesen R, Duchow A, Stiglbauer V, Ruprecht K, et al. SIGLEC1 (CD169): a marker of active neuroinflammation in the brain but not in the blood of multiple sclerosis patients. Sci Rep. 2021;11:10299.

202. Clancy RM, Halushka M, Rasmussen SE, Lhakhang T, Chang M, Buyon JP. Siglec-1 Macrophages and the Contribution of IFN to the Development of Autoimmune Congenital Heart Block. J Immunol. 2019;202:48–55.

203. Abdalla SA, Letarte M. Hereditary haemorrhagic telangiectasia: current views on genetics and mechanisms of disease. J Méd Genet. 2006;43:97.

204. Wang G, Wen B, Deng Z, Zhang Y, Kolesnichenko OA, Ustiyan V, et al. Endothelial progenitor cells stimulate neonatal lung angiogenesis through FOXF1-mediated activation of BMP9/ACVRL1 signaling. Nat Commun. 2022;13:2080.

205. Anderson KE, Bellio TA, Aniskovich E, Adams SL, Blusztajn JK, Delalle I. The Expression of Activin Receptor-Like Kinase 1 (ACVRL1/ALK1) in Hippocampal Arterioles Declines During Progression of Alzheimer’s Disease. Cereb Cortex Commun. 2020;1:tgaa031-.

206. Liu Y, Zhang L, Mei R, Ai M, Pang R, Xia D, et al. The Role of SliTrk5 in Central Nervous System. BioMed Res Int. 2022;2022:4678026.

207. Sun J, Shin DY, Eiseman M, Yallowitz AR, Li N, Lalani S, et al. SLITRK5 is a negative regulator of hedgehog signaling in osteoblasts. Nat Commun. 2021;12:4611.

208. Radhakrishna U, Nath SK, Vishweswaraiah S, Uppala LV, Forray A, Muvvala SB, et al. Maternal opioid use disorder: Placental transcriptome analysis for neonatal opioid withdrawal syndrome. Genomics. 2021;113:3610–7.

209. Hitchcock IS, Hafer M, Sangkhae V, Tucker JA. The thrombopoietin receptor: revisiting the master regulator of platelet production. Platelets. 2021;32:770–8.

210. Jeon GW. Pathophysiology, classification, and complications of common asymptomatic thrombocytosis in newborn infants. Clin Exp Pediatrics. 2021;

211. Hedstrom J, Sainio V, Kemppainen E, Haapiainen R, Kivilaakso E, Schroder T, et al. Serum complex of trypsin 2 and alpha-1 antitrypsin as diagnostic and prognostic marker of acute pancreatitis: clinical study in consecutive patients. BMJ. 1996;313:333.

212. Wang W, Wu L, Wu X, Li K, Li T, Xu B, et al. Combined analysis of serum SAP and PRSS2 for the differential diagnosis of CD and UC. Clin Chim Acta. 2021;514:8–14.

213. Marić I, Contrepois K, Moufarrej MN, Stelzer IA, Feyaerts D, Han X, et al. Early prediction and longitudinal modeling of preeclampsia from multiomics. Patterns. 2022;3:100655.

214. Ou R, Zhang M, Huang L, Flavell RA, Koni PA, Moskophidis D. Regulation of Immune Response and Inflammatory Reactions against Viral Infection by VCAM-1. J Virol. 2008;82:2952–65.

215. Kong D-H, Kim YK, Kim MR, Jang JH, Lee S. Emerging Roles of Vascular Cell Adhesion Molecule-1 (VCAM-1) in Immunological Disorders and Cancer. Int J Mol Sci. 2018;19:1057.

216. Leifsdottir K, Jost K, Siljehav V, Thelin EP, Lassarén P, Nilsson P, et al. The cerebrospinal fluid proteome of preterm infants predicts neurodevelopmental outcome. Frontiers Pediatrics. 2022;10:921444.

217. Leviton A, Fichorova R, Yamamoto Y, Allred EN, Dammann O, Hecht J, et al. Inflammation-related proteins in the blood of extremely low gestational age newborns. The contribution of inflammation to the appearance of developmental regulation. Cytokine. 2011;53:66–73.

218. Maloney JP, Gao L. Proinflammatory Cytokines Increase Vascular Endothelial Growth Factor Expression in Alveolar Epithelial Cells. Mediat Inflamm. 2015;2015:387842.

219. Reinders MEJ, Sho M, Izawa A, Wang P, Mukhopadhyay D, Koss KE, et al. Proinflammatory functions of vascular endothelial growth factor in alloimmunity. J Clin Invest. 2003;112:1655–65.

220. Voller SB, Chock S, Ernst LM, Su E, Liu X, Farrow KN, et al. Cord blood biomarkers of vascular endothelial growth (VEGF and sFlt-1) and postnatal growth: A preterm birth cohort study. Early Hum Dev. 2014;90:195–200.

221. The Royal College of Ophthalmologists. Treating Retinopathy of Prematurity in the UK. The Royal College of Ophthalmologists; 2022.

222. Collaco JM, McGrath-Morrow SA, Griffiths M, Chavez-Valdez R, Parkinson C, Zhu J, et al. Perinatal Inflammatory Biomarkers and Respiratory Disease in Preterm Infants. J Pediatrics. 2022;246:34-39.e3.

223. Oak P, Hilgendorff A. The BPD trio? Interaction of dysregulated PDGF, VEGF, and TGF signaling in neonatal chronic lung disease. Mol Cell Pediatrics. 2017;4:11.

224. Klerk DH, Plösch T, Verkaik-Schakel RN, Hulscher JBF, Kooi EMW, Bos AF. DNA Methylation of TLR4, VEGFA, and DEFA5 Is Associated With Necrotizing Enterocolitis in Preterm Infants. Frontiers Pediatrics. 2021;9:630817.

225. Monestier O, Blanquet V. WFIKKN1 and WFIKKN2: “Companion” proteins regulating TGFB activity. Cytokine Growth Factor Rev. 2016;32:75–84.

226. Kondás K, Szláma G, Trexler M, Patthy L. Both WFIKKN1 and WFIKKN2 Have High Affinity for Growth and Differentiation Factors 8 and 11. J Biol Chem. 2008;283:23677–84.

227. Szklarczyk D, Kirsch R, Koutrouli M, Nastou K, Mehryary F, Hachilif R, et al. The STRING database in 2023: protein–protein association networks and functional enrichment analyses for any sequenced genome of interest. Nucleic Acids Res. 2022;51:D638–46.
